# Follicle-stimulating hormone stimulates free radical generation without inducing substantial oxidative stress in human granulosa cells

**DOI:** 10.1093/hropen/hoaf007

**Published:** 2025-02-17

**Authors:** Nuan Lin, Koen C van Zomeren, Torsten Plosch, Naomi Hofsink, Teelkien van Veen, Hui Ting Li, Jiazhe Lin, Xiaoling Zhou, Henk Groen, Uwe J F Tietge, Astrid Cantineau, Romana Schirhagl, Annemieke Hoek

**Affiliations:** Department of Obstetrics and Gynecology, University Medical Center Groningen, University of Groningen, Groningen, The Netherlands; Department of Obstetrics and Gynecology, The First Affiliated Hospital of Shantou University Medical College, Shantou, China; Department of Obstetrics and Gynecology, University Medical Center Groningen, University of Groningen, Groningen, The Netherlands; Department of Obstetrics and Gynecology, University Medical Center Groningen, University of Groningen, Groningen, The Netherlands; Department of Obstetrics and Gynecology, University Medical Center Groningen, University of Groningen, Groningen, The Netherlands; Department of Obstetrics and Gynecology, University Medical Center Groningen, University of Groningen, Groningen, The Netherlands; Department of Obstetrics and Gynecology, University Medical Center Groningen, University of Groningen, Groningen, The Netherlands; Department of Biomedical Engineering, University Medical Center Groningen, University of Groningen, Groningen, The Netherlands; Department of Neurosurgery, The First Affiliated Hospital of Shantou University Medical College, Shantou, China; Center for Reproductive Medicine, Shantou University Medical College, Shantou, China; Department of Epidemiology, University Medical Center Groningen, University of Groningen, Groningen, The Netherlands; Division of Clinical Chemistry, Department of Laboratory Medicine, Karolinska Institutet, Stockholm, Sweden; Clinical Chemistry, Karolinska University Laboratory, Karolinska University Hospital, Stockholm, Sweden; Department of Obstetrics and Gynecology, University Medical Center Groningen, University of Groningen, Groningen, The Netherlands; Department of Biomedical Engineering, University Medical Center Groningen, University of Groningen, Groningen, The Netherlands; Department of Obstetrics and Gynecology, University Medical Center Groningen, University of Groningen, Groningen, The Netherlands

**Keywords:** follicle stimulating hormone, granulosa cell, reactive oxygen species, mitochondria, fluorescent nanodiamond (FND) magnetometry

## Abstract

**STUDY QUESTION:**

Does FSH induce free radical generation with substantial oxidative damage in human cumulus granulosa cells (cGCs) and mural granulosa cells (mGCs)?

**SUMMARY ANSWER:**

FSH of both physiological and supraphysiological concentrations induced free radical generation on subcellular levels, most notably in the mitochondria, while the elevated free radical load caused neglectable oxidative damage in both cGCs and mGCs.

**WHAT IS KNOWN ALREADY:**

FSH is fundamental for regulation of granulosa cell (GC) function and oocyte maturation, during which a physiological level of reactive oxygen species (ROS) is essential, while excessive amounts lead to oxidative damage. Potential adverse effects of high FSH doses on GCs may be mediated by ROS.

**STUDY DESIGN, SIZE, DURATION:**

This prospective experimental study included patients who attended a reproductive medicine center in 2023. cGC and mGC were separately isolated and brought into culture on the day of oocyte retrieval, 36 h after ovulation induction with recombinant hCG (250 mg). Recombinant FSH, at different concentrations, mimicking physiological (6 mIU/ml) and supraphysiological (60 and 600 mIU/ml) conditions, was applied (n = 4 in each group).

**PARTICIPANTS/MATERIALS, SETTING, METHODS:**

Women aged 20–35 years, undergoing ICSI with at least three follicles, were included. Quantum sensing of cGC and mGC free radicals, detected by either cytoplasm-located fluorescent nanodiamonds (FNDs) or mitochondria-targeted FNDs, was tracked for 2 h following FSH treatment in a magnetometry setup. Mitochondrial function analysis, as well as oxidative damage to DNA/RNA, lipids, and proteins, upon FSH exposure, was examined.

**MAIN RESULTS AND THE ROLE OF CHANCE:**

FSH-induced cytoplasmic and mitochondrial ROS increases in cGC and mGC (*P* < 0.01 in all concentrations after 2 h) while showing different patterns along time: cGC showed significantly larger cytoplasmic ROS change compared with mGC to physiological (*P* < 0.01) and supraphysiological (*P* < 0.05) concentrations of FSH. Significantly larger free radical changes were observed in the mitochondria compared to the cytoplasm in response to FSH (all concentrations in cGCs with *P* < 0.05; supraphysiological concentrations in mGCs with *P* < 0.05, *P* < 0.001, respectively) after 2 h. Mitochondrial basal respiration and ATP production were significantly increased upon FSH exposure to supraphysiological concentrations in both cGCs (*P* < 0.01) and mGCs (*P* < 0.05). However, no oxidative damage to GC DNA/RNA, proteins, or lipids was found upon FSH exposure at any concentration except elevated lipid peroxidation in the FSH group of 600 mIU/ml (*P* < 0.05).

**LARGE SCALE DATA:**

N/A.

**LIMITATIONS, REASONS FOR CAUTION:**

The GCs came from females of different biological backgrounds and were stimulated before oocyte and GC retrieval, thereby increasing the risk of variation. In addition, the effects of long-term FSH exposure as well as the effects of the FSH-induced ROS on the oocyte remain to be investigated.

**WIDER IMPLICATIONS OF THE FINDINGS:**

We demonstrate that FSH of both physiological and supraphysiological concentrations induces free radical generation at subcellular levels, most notably in the mitochondria, while the elevated free radical load causes neglectable oxidative damage in both cGCs and mGCs. Our results suggest that the ‘FSH Ootoxicity’ hypothesis would not seem to be mediated by ROS in human GCs.

**STUDY FUNDING/COMPETING INTEREST(S):**

This study is supported by the Abel Tasman Talent Program (ATTP) of the Graduate School of Medical Sciences of the University Medical Center Groningen/University of Groningen, The Netherlands, as well as an XS grant from NWO. Unrelated to the current work, A.H. is a member of an advisory board on the development and application of a lifestyle App for patients with infertility from Ferring Pharmaceutical Company, The Netherlands. R.S. is the founder of QT Sense B.V., who commercialize quantum sensing equipment. This article has no direct relation to the work of QT Sense B.V. The remaining authors have no conflicts of interest.

WHAT DOES THIS MEAN FOR PATIENTS? This study aims to answer the question of whether follicle stimulating hormone (FSH), a commonly administered hormone used to increase the chance of retrieving multiple oocytes during the IVF process, is potentially harmful to granulosa cells (GCs), which are cells in the ovaries that support oocyte development. Specifically, this study made use of GC samples from patients who were undergoing oocyte retrieval for IVF. We found that FSH, no matter whether at normal or higher levels, can lead to the production of free radicals, which are unstable molecules inside cells, especially in the mitochondria, which are the powerhouses of the cell. However, the amount of free radicals produced does not cause significant damage to granulosa cell components, including DNA/RNA, proteins, or lipids. This means that the theory which suggests that FSH might cause harm to the oocytes would not likely be due to the effects of the FSH-stimulated free radicals.

## Introduction

FSH is a glycoprotein hormone secreted by gonadotropic cells of the anterior pituitary lobe. FSH binds to follicle stimulating hormone receptor (FSHR), a member of the G-protein coupled receptor (GPCR) superfamily, thereby initiating a cascade of biochemical signaling events primarily in granulosa cells (GCs), the cells that surround and support the oocyte in the ovary. This contributes to a range of biological activities such as steroid hormone synthesis, cell proliferation, and survival, as well as induction of LH receptor expression, which regulates follicle function and oocyte development (Gloaguen *et al.*, 2011). GCs acquire FSH responsiveness at the preantral follicle stage before initiation of rapid proliferation upon FSH exposure (Richards, 1980). Consequently, an antrum is developed in the presence of FSH, and the GCs that originally enclose the oocyte differentiate into two distinct functional groups: the cumulus granulosa cells (cGCs) that are in immediate contact with the oocyte via gap junctions and the mural granulosa cells (mGCs) that line the wall of the follicular antrum (Eppig *et al.*, 1997). Not only functional heterogeneity but also differential responses to hormones between cGCs and mGCs have been reported (Khamsi and Roberge, 2001). For instance, hormone receptors, such as estrogen receptors, are differentially expressed between cGCs and mGCs in rodents as well as in humans (Kõks *et al.*, 2010; Wigglesworth *et al.*, 2015). Also, Khamsi and Roberge (2001) found that cGCs and mGCs proliferate differently in response to FSH. Thus, FSH plays a critical role in regulating the functions of cGC and mGC, thereby governing reproductive success.

However, growing attention has been paid to the potential adverse effects of high doses of FSH (Abdalla and Thum, 2004; Scheetz *et al.*, 2012; Clark *et al.*, 2022; Karl *et al.*, 2023). Bernstein *et al.* (2023) have recently proposed the ‘FSH Ootoxicity Hypothesis’, positing that high FSH is toxic to oocytes, especially in women of advanced reproductive age. Ovarian stimulation with excessive FSH was recently found to induce premature cGC expansion, thereby disrupting its function (Karl *et al.*, 2023). Bernstein *et al*. (2023) found that oocytes from aged mice are more susceptible to FSH induction of spindle misalignments. According to their hypothesis, ‘FSH Ootoxicity’ is also regarded as a source of low pregnancy success rates in naturally cycling females with high FSH, as well as in women undergoing ART (Bernstein *et al.*, 2023). These observations indicate that high-dose exogenous FSH may paradoxically impede optimal ART reproductive outcomes, while the mediators of such an effect are unclear.

A possible pathway through which supraphysiological FSH may lead to a detrimental effect on oocytes and surrounding GCs, and hence fertility, is an overabundance of reactive oxygen species (ROS) leading to oxidative stress. FSH stimulates GC proliferation. During this process, a continuous supply of high levels of ATP is required, while ROS is a natural by-product of this ATP generation. However, ROS generation does not uniformly translate into oxidative stress. ROS can act as important signaling molecules with no harm when their production is balanced by the cellular antioxidant defenses (Schieber and Chandel, 2014). Oxidative stress only arises when excessive ROS overwhelm these defenses (Schieber and Chandel, 2014). While a physiological level of ROS is essential for folliculogenesis, oocyte meiotic maturation, and ovulation (Hu *et al.*, 2001; Shkolnik *et al.*, 2011; Agarwal *et al.*, 2012), an excessive accumulation of free radicals can negatively impact oocyte quality, correlating with decreased female fertility (Pasqualotto *et al.*, 2004; Combelles *et al.*, 2009). Some evidence supports this hypothesis, as an *in vitro* study has demonstrated that the ROS level in mouse GCs stimulated with FSH for 24 h was significantly increased (Hoque *et al.*, 2021). In contrast, another study revealed that FSH protects mouse GCs from oxidative damage by repressing mitophagy (Shen *et al.*, 2016). A clinical study found that oxidative stress can decrease FSH-stimulated GC E2 production, which is predictive of the ovarian response to gonadotropin stimulation (Appasamy *et al.*, 2008). Hence, the effect of FSH on the regulation of ROS generation remains controversial and is insufficiently explored, which means that the study of FSH-induced ROS generation may be helpful to better understand potential effects behind ovarian stimulation cycles.

One of the reasons underlying this inconclusive and scarce knowledge could be that direct free radical detection in biological samples remains a great challenge due to their short lifespans and low abundance (Murphy *et al.*, 2022). Recently, we have applied a fluorescent nanodiamond (FND)-based method, allowing temporospatial measurement of free radical change upon menadione in human GCs (Lin *et al.*, 2023). Due to their advantages of stable fluorescence and biocompatibility, FNDs are developed for real-time tracking and labeling of free radicals (Morita *et al.*, 2023). Based on negatively charged nitrogen-vacancy (NV^−^) defects (Hsiao *et al.*, 2016; Hui *et al.*, 2017; Panwar *et al.*, 2019; Sigaeva *et al.*, 2022), which can ‘feel’ magnetic noise from free radicals and convert it into an optical signal, FND relaxometry offers a way for direct free radical measurement in real-time and on a subcellular level. Since the current knowledge on FSH-associated ROS effects in GCs relies on indirect measurements of ROS (Shen *et al.*, 2016; Hoque *et al.*, 2021), a direct temporospatial tracking of ROS following FSH exposure is relevant and informative.

Therefore, in the current study, we aimed to investigate whether physiological and supraphysiological levels of FSH induce free radical generation in human cGCs and mGCs on a subcellular level in real-time. Furthermore, we hypothesized that supraphysiological FSH levels lead to an overabundance of free radicals, resulting in oxidative damage to GC.

## Materials and methods

### Patient recruitment and sample collection

This study was carried out at the Department of Reproductive Medicine, University Medical Centre Groningen, The Netherlands, between March 2023 and November 2024. Primary GCs were obtained from follicles of 38 healthy females undergoing oocyte retrieval for IVF. Patients were stimulated with recombinant FSH dosages ranging from 150 to 225 IU per day for 10–14 days. The inclusion criteria encompassed women aged 20–35 years, possessing a minimum of three follicles with a diameter exceeding 18 mm on the day of follicle triggering, and undergoing ICSI. Exclusion criteria were chromosomal abnormalities or hydrosalpinx.

Ethical considerations were cautiously addressed, with approval from the Institutional Review Board sought and subsequently waived, as the study employed anonymized waste material, specifically, GCs that are routinely available post-oocyte retrieval. All participating patients provided informed consent, with their materials processed anonymously. Notably, patients consented to the utilization of their GCs, which, otherwise destined for disposal as waste material, were repurposed for research.

Follicular fluid containing mGCs was collected during oocyte retrieval, which took place 36 h after injection of 250 µg recombinant hCG (Ovitrelle^®^, Merck Serono, Modugno, Italy). cGC clusters were mechanically dissected from the cumulus–oocyte complex and stored in G-MOPS (Vitrolife, Gothenburg, Sweden). Both cell samples were brought to a cell culture hood for further experiments.

### Cell isolation and culture

The isolation of cGCs and mGCs followed established protocols (Lin *et al.*, 2023). For cGC isolation, the cell clusters were gently disaggregated by pipetting. For mGC isolation, the follicular fluid containing mGCs was centrifuged at 600 g for 7 min to pellet the cells. The resulting cell pellet was resuspended in phosphate-buffered saline (PBS). To remove contaminating red blood cells, mGC pellets were layered over a 40% Percoll™ gradient (Cat. No. 10607095, Fisher Scientific, Waltham, MA, USA), and then cells were collected from the interface after centrifugation. To dissociate cell clusters and obtain single-cell suspensions, the cells were strained through a Falcon 40 µm strainer (Cat. No. 352340, Corning, NY, USA) after being dispersed by trypsinization. For experiments requiring plating with specific cell densities, cGCs and mGCs were seeded accordingly, before being cultured in Dulbecco’s modified Eagle medium/nutrient mixture F-12 (DMEM/F12) (Cat. No. 11320033, Life Technologies, Carlsbad, CA, USA) supplemented with 10% fetal calf serum (FCS) and 1% penicillin–streptomycin–amphotericin B at 37°C and 5% CO_2_. For T1 measurements and confocal microscopy, cells were plated into 35 mm culture dishes (CELLview™ Culture dish, non-treated, four compartments, glass bottom, Greiner Bio-One, Item No. 627870, Monroe, NC, USA) at a density of 4000 cells per compartment. For mitochondrial analysis, 15 000 cells per well were seeded in the Seahorse plates from the Seahorse XF Cell Mito Stress Test Kit (103015-100, Agilent Technologies, Santa Clara, CA, USA). For assays such as 2′,7′-dichlorodihydrofluorescein diacetate (DCFH-DA) and 3-[4,5-dimethylthiazol-2-yl]-2,5 diphenyl tetrazolium bromide (MTT), cells were plated into 96-well plates (Tissue Culture-Treated, Flat-Bottom with lid, Corning) at a density of 10 000 cells per well. Following seeding, the culture medium was refreshed after 48–72 h to maintain cell viability and function. Cells were utilized in experiments within 1 week to ensure optimal performance and consistency.

### Materials

Recombinant FSH was from a GONAL-f^®^ pre-filled pen that is routinely used in the reproductive clinic. FNDs with a hydrodynamic diameter of 70 nm, each containing more than 300 NV centers, were procured from Adámas Nanotechnologies (Raleigh, NC, USA) for their suitability in T1 measurements (Perona Martínez *et al.*, 2020). These FNDs are widely employed in the field and have been characterized in previous studies (Hemelaar *et al.*, 2017; Ong *et al.*, 2018; Su *et al.*, 2019). For functionalization, the anti-voltage-dependent anion channel isoform 2 (anti-VDAC2) antibody ([C2C3], C-term, catalog no. GTX104745) obtained from GeneTex (Huissen, The Netherlands) was used. Following established protocols (Nie *et al.*, 2021), two kinds of particles were freshly prepared prior to use: uncoated FNDs (bare-FNDs), which are expected to be in the cytoplasm at the timing of the measurement, and mitochondria-targeting FNDs, coated with physically adsorbed anti-VDAC2 antibodies which bind to voltage-dependent anion channel isoform 2 (aVDAC2-FNDs) targeting the mitochondrial outer membrane (Nie *et al.*, 2021; Lin *et al.*, 2023).

Menadione (Cat. No. M5625-25G) was purchased from Merck. 8-hydroxy-2-deoxyguanosine (8-OHdG) antibody (15A3) (mouse, Cat. No. sc-66036) and translocase of the outer mitochondrial membrane 20 (Tom20) antibody (rabbit, Cat. No. sc-11415) were purchased from Santa Cruz Biotechnology (Dallas, TX, USA). Donkey-α-rabbit Red-X secondary antibody (Jackson ImmunoResearch, Cat. No. 711-295-152, West Grove, PA, USA) and Goat anti-Mouse FITC Secondary Antibody (Cat. No. F-2761, Abcam, Cambridge, UK) were used. A Cellular ROS Assay Kit (DCFDA/H2DCFDA, ab113851) was purchased from Abcam.

### ROS assay

Both cGCs and mGCs were, respectively, incubated with FSH of different concentrations (6, 60, and 600 mIU/ml) or with DMEM/F12 only for 2 h. Menadione (10 µM) served as a positive control of intracellular ROS induction. Two hours later, cells were incubated with DCFDA according to the manufacturer’s protocol. All the fluorescence intensities were measured by a plate reader (BioTek, Santa Clara, CA, USA) at Ex/Em = 485/535 nm prevented from light. Cells without staining with DCFDA were recorded for background subtraction.

### Cell viability assay

Following the instructions of the manufacturer, the viabilities of cGCs and mGCs were measured by an MTT assay which, in principle, detects nicotinamide adenine dinucleotide phosphate (NAD(P)H)-dependent oxidoreductase activity. cGCs and mGCs were, respectively, incubated with different concentrations of FSH as mentioned above or HCl for 2 h. HCl (0.1 M) was employed as a positive control to induce cell death. Absorbance was measured using a plate reader (Bio Tek) at 570 nm. Experiments were performed in three replicates. Samples were normalized against the mean absorbance value of the control group.

### Relaxometry measurements

Following incubation with 1 µg/ml of either bare-FNDs or aVDAC2-FNDs for 24 h, cells were initially washed with PBS before replacing the PBS with DMEM/F12 medium. Subsequently, T1 (relaxometry) measurements were conducted utilizing a laser pulsing sequence within a custom-made magnetometry setup, which is essentially a modified confocal microscope (Morita *et al.*, 2020; Wu *et al.*, 2022).

In a typical T1 measurement, NV centers are pumped into the bright ms = 0 state of the ground state, followed by probing after various dark times to assess whether the NV centers remain in this state or return to the darker equilibrium between ms = 0 and ms = ±1. In the presence of free radicals, this process occurs faster. Measuring this time can be used to quantify free radical generation (Rollo *et al.*, 2021). To extract the magnetic noise level from these plots, we used a double exponential fit of the form:

(1) PL(τ)=I inf (1+Ca e−τ/Ta+C b e−τ/Tb).

This fit is different from the single exponential fits, which are used for single NV center measurements. After making the observation that the single exponential model does not represent the data well in ensembles in nanodiamonds, this model was determined empirically. This fit considers that there are different NV centers with different T1 values within each particle. While both constants respond to changes in magnetic noise, the longer constant is more sensitive to changes. This was found earlier by measuring different known concentrations and observing how the different constants respond. Thus, to quantify free radical generation, we used the longer time constant Tb, which we call T1 (Perona Martínez *et al.*, 2020). A detailed discussion of the bi-exponential model as well as comparison with other models can be found in (Vedelaar *et al.*, 2023). This measurement reveals a signal that is equivalent to T1 in conventional MRI. However, since NV centers only detect their local environment (up to a few tens of nm), this method offers nanoscale resolution (Mamin *et al.*, 2013; Staudacher *et al.*, 2013).

The laser utilized in our experiments was a 532 nm laser emitting at 50 µW at the sample location, measured during continuous illumination. The measurement sequence comprised 5 µs long laser pulses separated by variable dark times τ ranging from 0.2 to 1000 µs. To execute the pulsing sequence, an acousto-optical modulator (Gooch & Housego, model 3350-199, Ilminster, UK) and an oil objective (×100) (Olympus, UPLSAPO 100XO, NA 1.40, Landsberg am Lech, Germany) were employed.

During the experiment, FND particles were identified and selected based on the following specific criteria under a bright field camera (Thorlabs): (i) the FND was positioned inside a cell; (ii) the brightness was ∼3 million photon counts/s; and (iii) the fluorescence exhibited stability, indicating that any bleaching structures observed were background fluorescence rather than FNDs.

Subsequently, an FND of each type inside the cell was selected and tracked for 120 min, and the free radical generation was measured via time-dependent T1. Prior to each measurement, it was verified once again that the particle met the criteria for being an FND by identifying its photon count and stable fluorescence.

All T1 measurements were performed at 37°C and under ambient air to ensure consistency throughout the experiment.

### Mitochondrial respiration measurements

The real-time oxygen consumption rate (OCR) of the cells was measured using an extracellular flux analyzer (Seahorse XF Pro, Agilent Technologies, Palo Alto, CA, USA). First, cells were seeded in XF cell culture microplates and induced. The culture medium was then replaced with non-buffered XF basal medium (#103575-100, Agilent Technologies, XF DMEM, pH 7.4) supplemented with 10 mM of glucose, 0.25 mM of NaOH, and 2 mM of glutamine, and the cells were allowed to degas in a non-CO_2_ incubator at 37°C for 1 h prior to the measurement. The cellular OCR was then determined before and after sequential injection of oligomycin (2.5 µM), dinitrophenol (50 µM), and rotenone/antimycin A (2/4 µM). The averaged OCR was monitored using the extracellular flux analyzer in a cycle consisting of mixing (2 min) and measuring (2 min). This cycle was repeated four times following each reagent injection. Basal respiration, maximal respiration, H^+^ (proton) leak, ATP production, non-mitochondrial oxygen consumption, and spare respiratory capacity were determined as follows:

Basal respiration=(last rate measurement before oligomycin injection)−(non−mitochondrial oxygen consumption)

 

Maximal respiration=(maximal rate measurement after dinitrophenol injection)−(non−mitochondrial oxygen consumption)

 

$$H^{+}\left(\text{proton}\right)\;\text{leak}=\text{minimum}\;\text{rate}\;\text{measurement}\;\text{after}\;\text{oligomycin}\;\text{injection}-\left(\text{non}-\text{mitochondrial}\;\text{oxygen}\;\text{consumption}\right)$$

 

ATP production=(last rate measurement before oligomycin injection)−(minimum rate measurement after oligomycin injection)

 

Non−mitochondrial oxygen consumption=minimum rate measurement after rotenone/antimycin A injection

 

Spare respiratory capacity=maximal respiration−basal respiration

### Confocal microscopy

After 2-h treatments with FSH of different concentrations or menadione (10 µM), cGCs and mGCs were fixed and underwent specimen preparation as described before (Lin *et al.*, 2023). Cells were then incubated with mouse-8-OHdG antibody (15A3) and rabbit-Tom20 antibody for 3 h at room temperature or overnight at 4°C. 8-OHdG antibody detects 8-OHdG (8-hydroxy-2′-deoxyguanosine, 8-hydroxyguanine, and 8-hydroxyguanosine), which are all markers of oxidative damage to RNA and DNA. Tom20 was stained for visualization of the mitochondria. Next, cells were incubated with staining solution consisting of 4 µg/ml 4′,6-diamidino-2-phenylindole (DAPI), 1:200 of goat-α-mouse Alexa 488 secondary antibody, and donkey-α-rabbit Alexa Fluor™ Plus 555 secondary antibodies at room temperature protected from light. Images were taken using a 63× 1.30 Glycerol objective in a Leica SP8X DLS confocal microscope (Leica Microsystems, Wetzlar, Germany) with a 405-nm laser to detect DAPI, a 488-nm laser to measure 8-OHdG, and a 561-nm laser to detect Tom20. All images were processed using the Fiji software.

### Lipid peroxidation assay

cGCs and mGCs were treated with FSH of different concentrations or 10 µM menadione for 2 h. Lipid peroxidation levels were measured as previously described (Zhang *et al.*, 2018). Menadione served as a positive control. Briefly, cells were incubated with 5 µM BODIPY 581/591 C11 dye (D3861, Invitrogen, Waltham, MA, USA). After incubation for 30 min at 37°C, cells were washed with PBS twice before flow cytometry analysis using a Quanteon Flow Cytometer System (Agilent Technologies). Data acquisition and analyses were performed using NovoExpress software.

### Protein carbonylation measurement

Protein carbonyl concentration of GCs was determined by using a Protein Carbonyl Content Assay Kit (MAK-094, Sigma-Aldrich, Darmstadt, Germany) according to the manufacturer’s instructions. The protein carbonyl assay is based on measuring stable carbonyl groups formed as a result of ROS oxidizing proteins. To reach a protein amount of 0.5 mg/well required by the assay kit, cGCs and mGCs were pooled before being treated with FSH of different concentrations or a positive control (10 µM menadione) for 2 h. The concentration of total protein was determined by a Bicinchoninic Acid Protein Assay Kit (BCA1, Sigma-Aldrich) according to the manufacturer’s instructions. The amount of protein carbonyl in each sample was reported as nmol protein carbonyl per mg of total protein.

### Statistical analysis

Quantitative data were expressed as the mean ± SD. Statistical analyses were performed using the R programming language. Significance testing was conducted utilizing either one-way ANOVA followed by a Tukey post hoc test or a *t*-test, as specified in the legend of each figure. Generalized estimating equation (GEE) models were employed to assess normalized T1 related to FSH concentration. Statistical significance was defined as follows: * for *P* < 0.05, ** for *P* < 0.01, *** for *P* < 0.001, and **** for *P* < 0.0001. All the exact *P*-values are shown in Supplementary Table S1.

## Results

**Figure 1.**
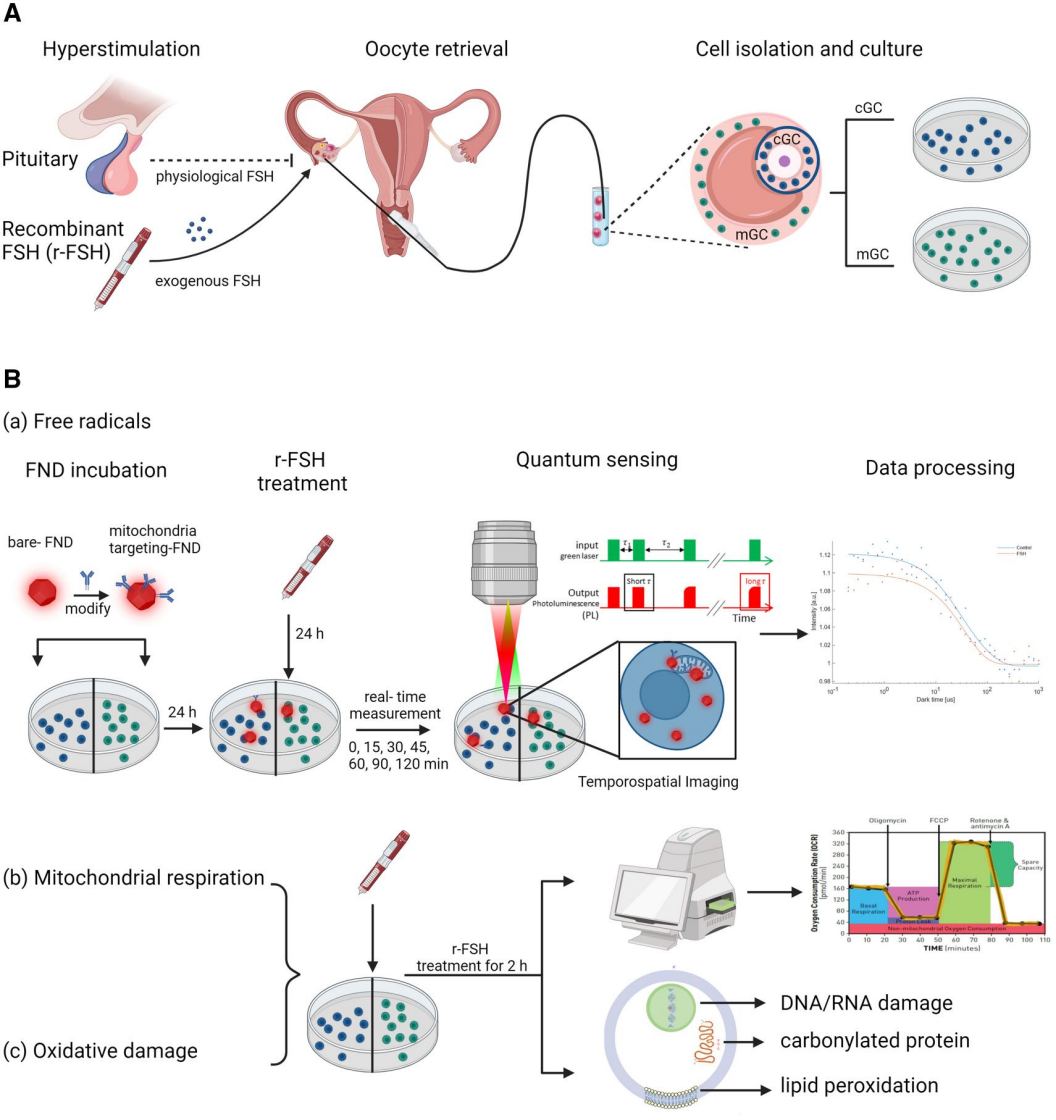
**Schematic summary of the experiments in this article**. (**A**) Oocyte retrieval was performed in females after ovarian stimulation for infertility treatment. cGCs and mGCs from preovulatory follicles were separately isolated and cultured. (**B**) Free radical, mitochondrial respiration, and oxidative damage in cGCs and mGCs were measured following FSH treatment. (**a**) Relaxometry was applied to probe temporospatial free radical change after FSH treatment in cGCs and mGCs by using bare FND and mitochondria-targeting FNDs. (**b**, **c**) Mitochondrial respiration and potential oxidative damage to DNA/RNA, proteins, and lipids of cGCs and mGCs were measured after FSH treatment for 2 h. Part of the figures were created with BioRender.com. cGCs: cumulus granulosa cells; mGCs: mural granulosa cells; FND: fluorescence nanodiamond.

Figure 1 shows a schematic summary of the experiments that were conducted in this study on cGCs and mGCs isolated from preovulatory follicles of females during an ICSI procedure. High purity of both cGCs and mGCs after isolation has been confirmed in our previous study (Lin *et al.*, 2023).

### FSH exposure of different concentrations increases intracellular ROS levels while not affecting cGC and mGC viability

**Figure 2.**
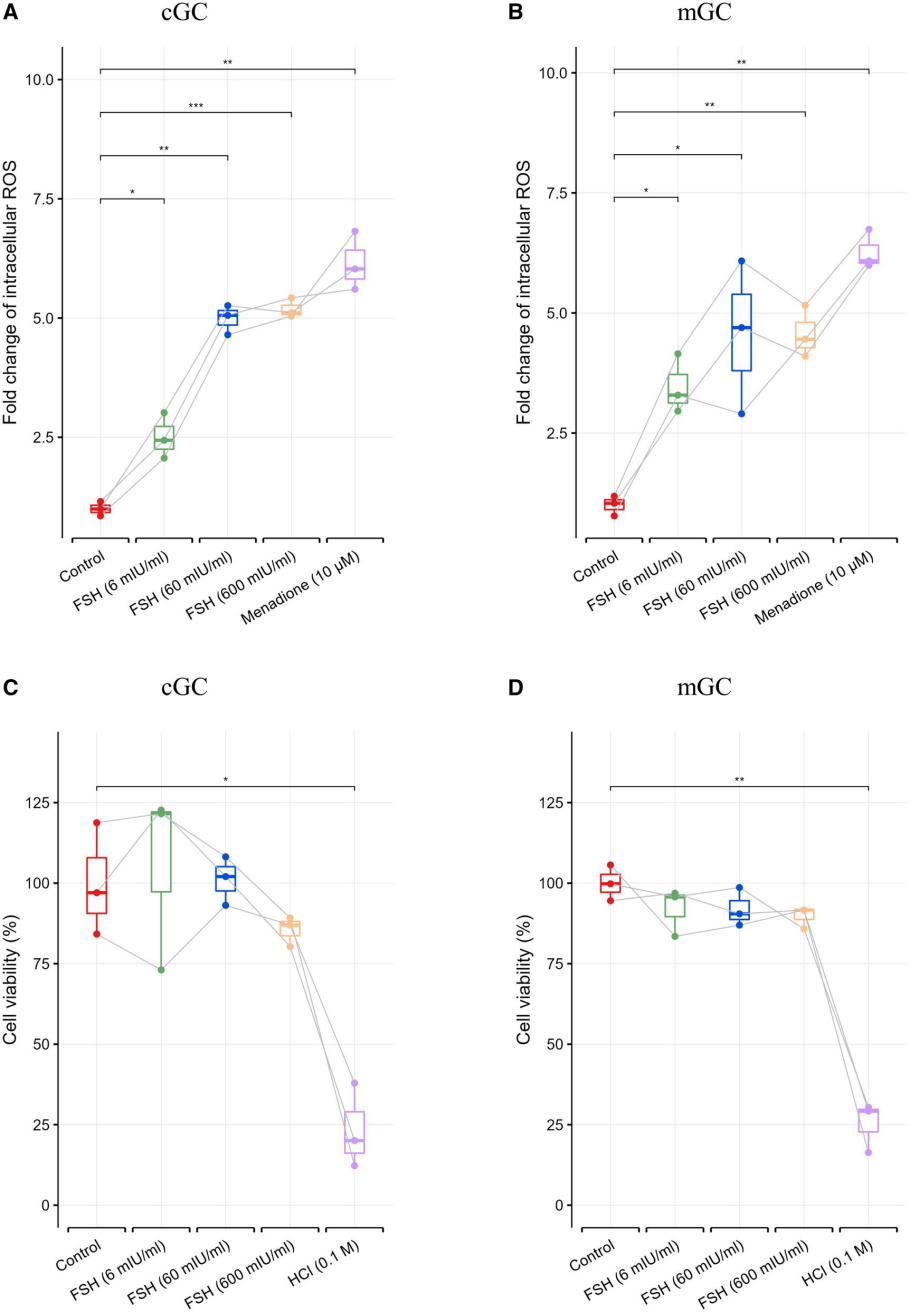
**Effects of FSH with different concentrations on intracellular reactive oxygen species and cell viability**. DCFH-DA assays show intracellular ROS generation after incubation with FSH of different concentrations (6, 60, and 600 mIU/ml) and menadione (10 µM) for 2 h in cGCs (**A**) and mGCs (**B**). Cell viabilities were determined by MTT assays after incubation with FSH of different concentrations (6, 60, and 600 mIU/ml) and HCl (0.1 M), respectively, in cGCs (**C**) and mGCs (**D**). One hundred percent represents a control without any stimuli exposure. The experiment was repeated for cells from three patients, and error bars represent the SDs. The data were analyzed by using paired *t*-test in comparison to the control (untreated) groups. *P < 0.05, **P < 0.01, ***P < 0.001. ROS: reactive oxygen species; DCFH-DA: 2′,7′-dichlorodihydrofluorescein diacetate; MTT: 3-[4,5-dimethylthiazol-2-yl]-2,5 diphenyl tetrazolium bromide; cGCs: cumulus granulosa cells; mGCs: mural granulosa cells.

To test whether intracellular ROS levels are increased by recombinant FSH, a DCFH-DA assay was conducted. Exposure to FSH of all tested concentrations for 2 h increased intracellular ROS levels in both cGCs and mGCs, resulting in significant differences between the control and FSH groups (*P* < 0.05, Fig. 2A and B). To exclude the possibility that higher overall ROS levels were the result of increased cell numbers due to the potential proliferation-inducing capacity of FSH, MTT assays were performed. There were no differences between the control and the FSH groups (*P* > 0.05, Fig. 2C and D), suggesting that FSH treatment for 2 h does not stimulate or inhibit cell proliferation. Thus, the increase in DCFH intensity originates from the increase in the intracellular ROS *per se*.

### Cytoplasmic and mitochondrial free radical levels were increased following FSH exposure in cGCs and mGCs

**Figure 3.**
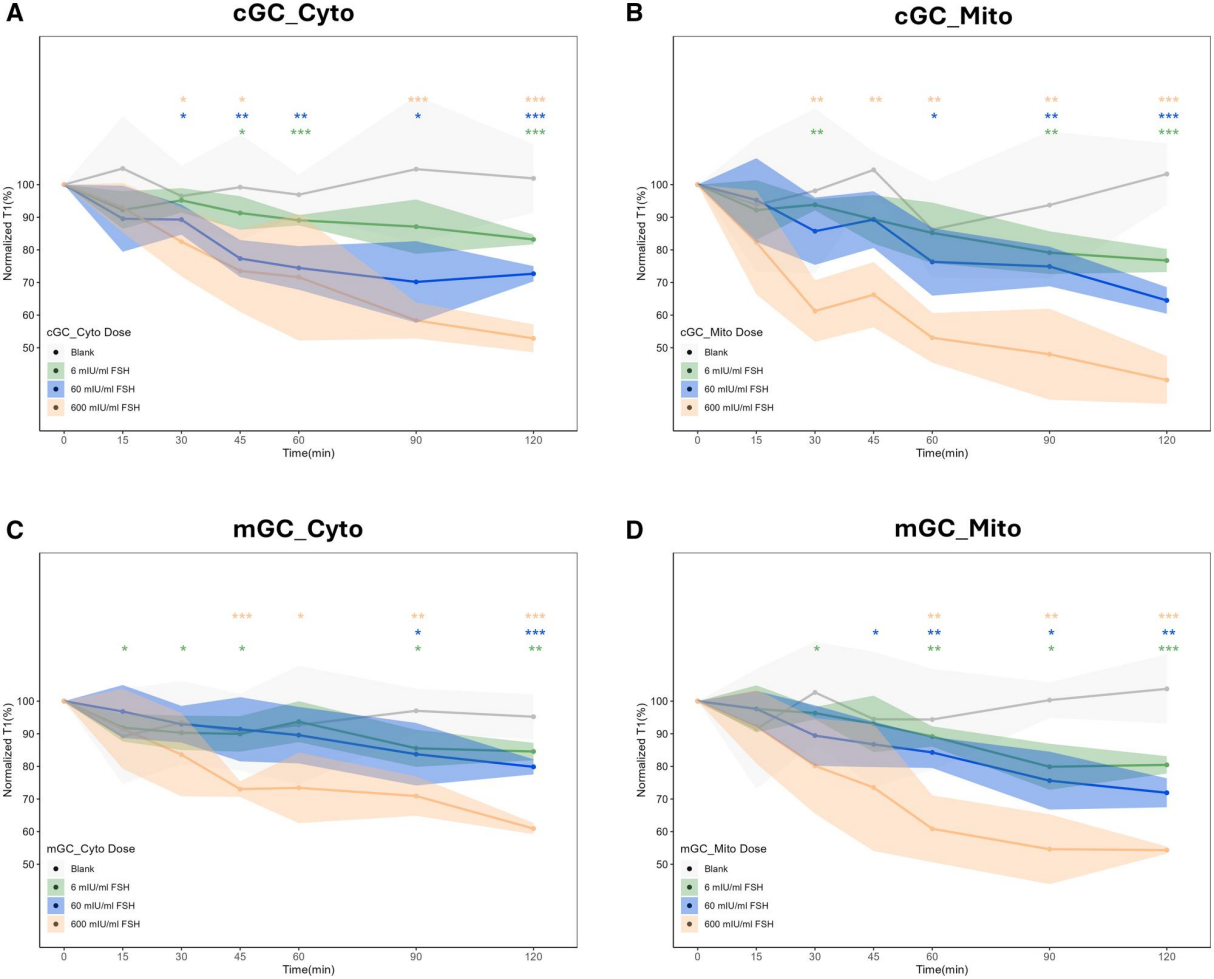
**Free radical detection by T1 relaxation in different timepoints in cGCs and mGCs with or without FSH treatment**. Timepoints: 0, 15, 30, 45, 60, 90, 120 min in cGCs (**A**, **B**) and mGCs (**C**, **D**). T1 data obtained from bare FND (A) and mitochondria-targeting FNDs (B) incubated with or without FSH-stimulated cGCs post-FSH treatment of different concentrations. T1 data obtained from bare FND (C) and mitochondria-targeting FNDs (D) incubated w/wo FSH-stimulated mGCs post-FSH treatment of different concentrations. Average of independent normalized T1 curves for each group. The data were analyzed by using paired *t*-test in comparison to the control (0 min) groups and presented as mean ± SD. N = 4 for each group, *P < 0.05, **P < 0.01, ***P < 0.001. cGCs: cumulus granulosa cells; mGCs: mural granulosa cells; FND: fluorescence nanodiamond.

Characterization, uptake, and localization of bare-FNDs and aVDAC2-FNDs have been shown in our previous study (Lin *et al.*, 2023). Figure 3 shows such measurements with bare-FNDs and mitochondria-targeting FNDs. As evident from Fig. 3, FSH exposure of different concentrations (0, 6 mIU/ml, 60 mIU/ml, and 600 mIU/ml) in both kinds of GCs leads to a decrease in T1 values. This corresponds to an increase in free radical concentration near the nanodiamond sensor. Raw T1 data of each patient are shown in Supplementary Figs S1, S2, S3, and S4. A calibration for OH* radicals at known concentrations was obtained from previous work to estimate the radical concentrations for the T1 values (Perona Martínez *et al.*, 2020). All T1 values with their distributions are shown in Supplementary Fig. S5. To exclude the possibility that T1 values can be affected by the surrounding environment, such as pH (Fujisaku *et al.*, 2019), during the 120-min measurement, blank groups without FSH exposure (gray lines) were set for T1 measurements in both kinds of cells as well as both FND variants. Consistent with a previous study (Nie *et al.*, 2021), no significant differences were found at any of the six timepoints compared to baseline.

Normalized T1 reduction curves in response to different FSH concentrations over time in cGC cytoplasm and mitochondria are shown in Fig. 3A and B, respectively. Similarly, normalized T1 reduction curves of different concentrations over time in mGC cytoplasm and mitochondria, measured by bare-FNDs and mitochondria-targeting FNDs, are given in Fig. 3C and D, respectively. From the shapes and slopes of the curves, it is obvious that free radical concentrations in the groups treated with FSH of 600 mIU/ml change most drastically over time, followed by those treated with 60 mIU/ml of FSH. Free radical loads in the groups treated with 6 mIU/ml of FSH showed the smallest reductions over time. GEE demonstrated that the highest sensitivity to FSH was observed in the mitochondria of cGC (Supplementary Tables S2, S3, S4, and S5).

Normalized T1 values at selected time points (15, 30, 45, 60, 90, and 120 min) were compared with those at 0 min before the addition of FSH. In both kinds of cells and both preparations of FNDs, normalized T1 showed significant differences at 120 min compared to 0 min (all *P*s < 0.01) (Fig. 3A–D).

### Free radical changes upon FSH exposure differ across cellular compartments and GC subtypes

**Figure 4.**
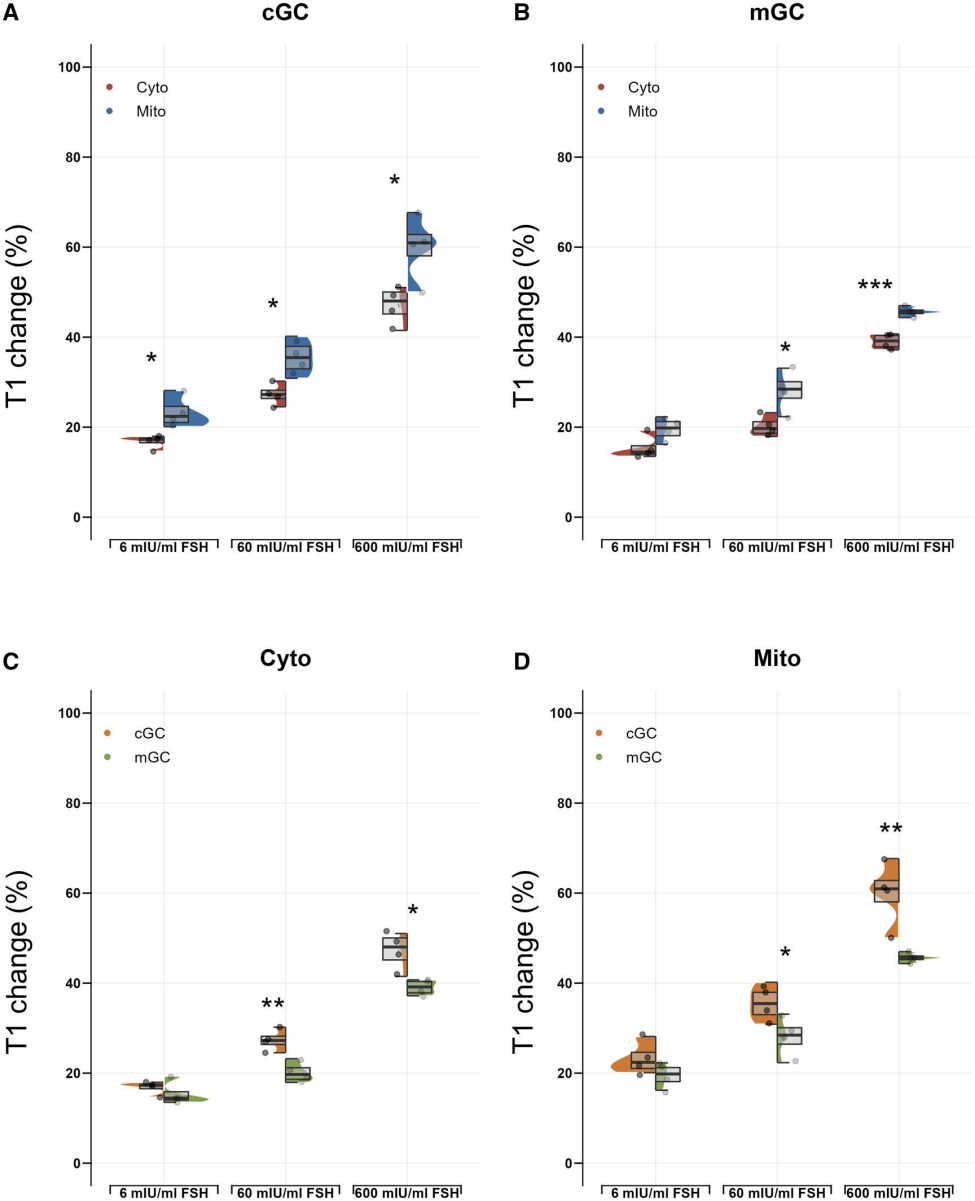
**Differences in free radical changes between cGCs and mGCs, cytoplasm, and mitochondria in response to FSH of different concentrations after 2 h**. Percentages of T1 changes from all the bare-FNDs and aVDAC2-FNDs were compared to show the differences between cytoplasm and mitochondrial free radical changes at 120 min when compared to the control in cGCs (**A**) and mGCs (**B**). (**C**) Percentages of T1 changes measured by the bare-FNDs in cGCs and mGCs were compared to show the differences between cGC and mGC cytoplasm free radical changes at 120 min when compared to the control. (**D**) Percentages of T1 changes measured by the mitochondria-targeting FNDs in cGCs and mGCs were compared to show the differences between cGC and mGC mitochondrial free radical changes at 120 min when compared to the control. Significance was tested by using a *t*-test. N = 4 for each group. *P < 0.05, **P < 0.01, ***P < 0.001. cGCs: cumulus granulosa cells; mGCs: mural granulosa cells; FND: fluorescence nanodiamond; aVDAC2: voltage-dependent anion channel isoform 2.

To investigate whether FSH exposure induces different free radical changes in the cytoplasm and the mitochondria as well as between the two GC subtypes, we compared the percentage of T1 reduction at 120 mins after exposure to FSH of each concentration (6, 60, and 600 mIU/ml). In cGCs, the percentages of T1 change were significantly greater in the mitochondria compared to the cytoplasm in all groups (*P* < 0.05) (Fig. 4A), suggesting that free radical changes were consistently more obvious in the mitochondria. Similar results were also observed in mGCs: the percentages of T1 changes were significantly greater in the mitochondria (*P* < 0.05 in 60 mIU/ml group and *P* < 0.001 in 600 mIU/ml group) (Fig. 4B).

Using bare-FNDs which measure free radical levels in the cytoplasm, we found no significant differences between cGCs and mGCs in the FSH group of 6 mIU/ml (*P* > 0.05) (Fig. 4C). However, in the FSH groups of 60 and 600 mIU/ml, the percentage of T1 changes in cGCs was significantly greater compared to that in mGCs (*P* < 0.01 in the FSH group of 60 mIU/ml and *P* < 0.05 in the FSH group of 600 mIU/ml) (Fig. 4C). These results suggest that although the cytoplasmic free radical levels are comparable between cGCs and mGCs under physiological concentrations of FSH, more free radicals were generated in cGC cytoplasm compared to mGC cytoplasm upon supraphysiological FSH exposure. A similar relative T1 change was observed when aVADC2-FNDs were used to measure the mitochondrial free radicals. Supraphysiological FSH stimulation (60 and 600 mIU/ml) induced significantly more free radical generation in cGCs. However, comparable free radical production between cGCs and mGCs was found after physiological FSH stimulation (6 mIU/ml) (*P* < 0.05) (Fig. 4D). These results indicate that although cGCs and mGCs respond to physiological levels of FSH similarly with respect to free radical generation, they show different responses to supraphysiological levels of FSH.

### Bioenergetic profiling of aerobic respiratory capacity of cGCs and mGCs after FSH exposure

**Figure 5.**
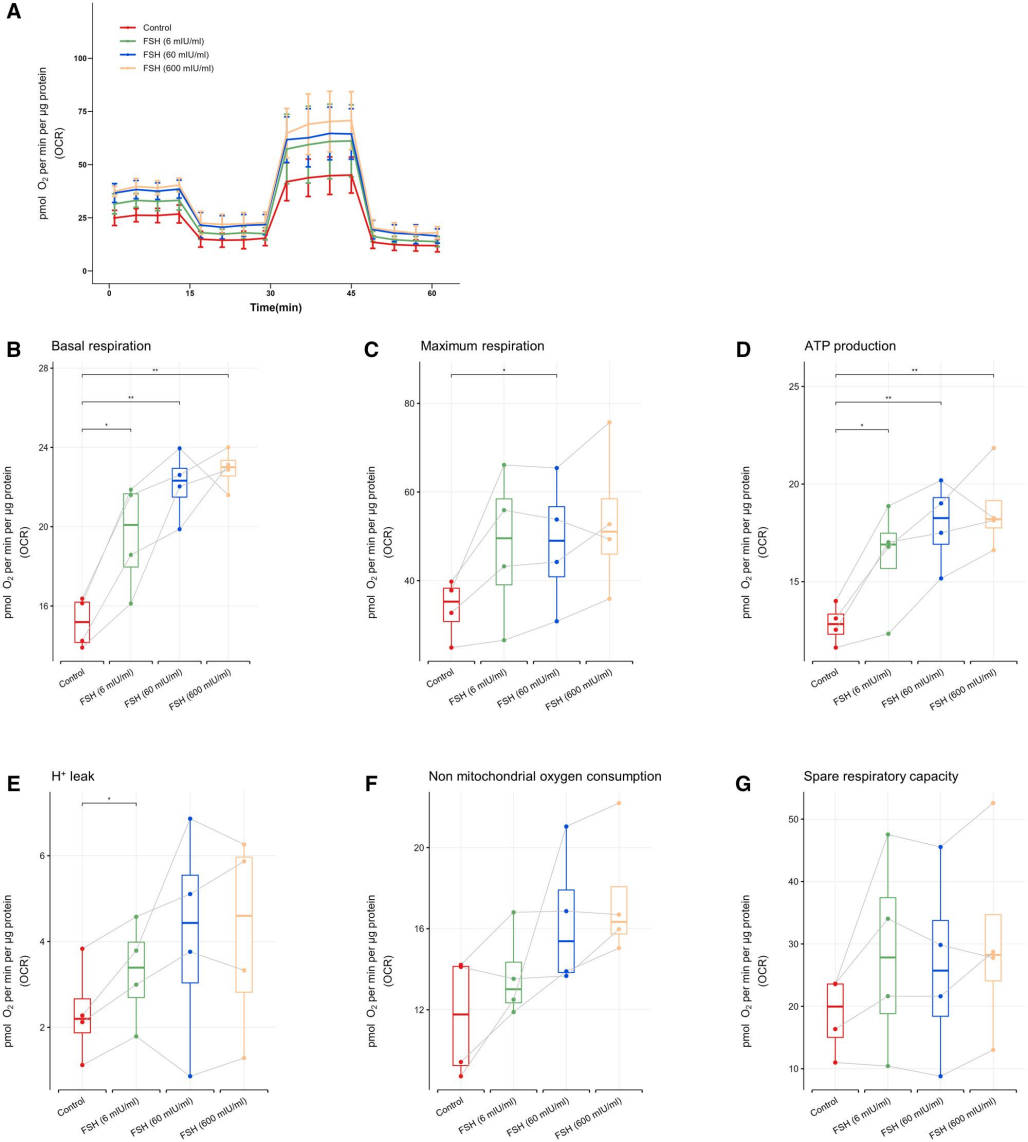
**Comparison of mitochondrial respiration of cGCs after FSH treatment of different concentrations for 2 h**. (**A**) The OCR of cGCs was measured by a Seahorse XFe96 Analyzer. Several parameters related to mitochondrial stress and aerobic respiratory capacity, including (**B**) basal respiration, (**C**) maximum respiration, (**D**) ATP production, (**E**) H^+^ leak, (**F**) non-mitochondrial respiration, and (**G**) spare respiratory capacity. Not all *y*-axes start at 0. The data were analyzed by using paired *t*-test in comparison to the control (untreated) groups and presented as mean ± SD. N = 4 for each group, *P < 0.05, **P < 0.01. OCR: oxygen consumption rate; cGCs: cumulus granulosa cells; mGCs: mural granulosa cells.

It is generally believed that the main source of intracellular free radicals is mitochondrial respiration (Kowalczyk *et al.*, 2021). To investigate whether mitochondrial respiration is also regulated by FSH, mitochondrial function analysis was performed after FSH exposure for 2 h. OCRs were measured in cGCs both without and with FSH exposure to varying concentrations of FSH; measurements were taken every 4 min within a total of 60 min (Fig. 5A). OCR plotting curves showed that the mitochondrial respiration after 2 h of FSH exposure was generally higher compared to the control group in cGCs. Specifically, basal respiration (Fig. 5B) and ATP production (Fig. 5D) in cGCs significantly increased 2 h after exposure to FSH at all concentrations (*P* < 0.05 in the group of 6 mIU/ml, *P* < 0.01 in both the groups of 60 and 600 mIU/ml). Compared to the control, the maximal respiration (Fig. 5C) and H^+^ leak (Fig. 5E) were also increased following exposure to 60 and 6 mIU/ml of FSH, respectively (*P* < 0.05). No significant differences were observed among the groups in terms of non-mitochondrial oxygen consumption (Fig. 5F), and spare respiratory capacity (Fig. 5G).

**Figure 6.**
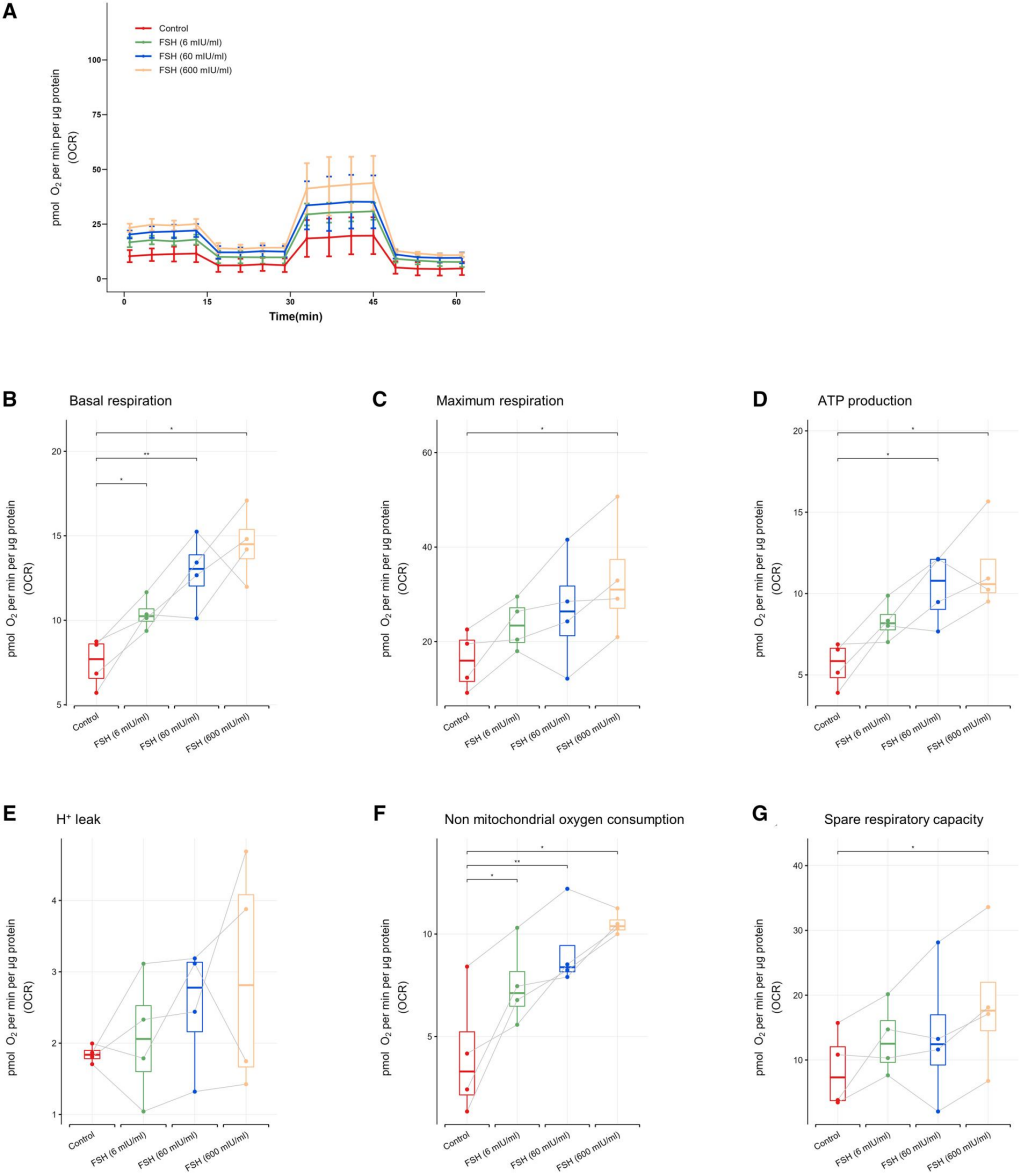
**Comparison of mitochondrial respiration of mGCs after FSH treatment of different concentrations for 2 h**. (**A**) The OCR of mGCs was measured by a Seahorse XFe96 Analyzer. Several parameters related to mitochondrial stress and aerobic respiratory capacity, including (**B**) basal respiration, (**C**) maximum respiration, (**D**) ATP production, (**E**) H^+^ leak, (**F**) non-mitochondrial respiration, and (**G**) spare respiratory capacity. Not all *y*-axes start at 0. The data were analyzed by using paired *t*-test in comparison to the control (untreated) groups and presented as mean ± SD. N = 4 for each group. *P < 0.05, **P < 0.01. OCR: oxygen consumption rate; cGCs: cumulus granulosa cells; mGCs: mural granulosa cells.

Similar increases in mitochondrial respiration after FSH exposure compared to the control were also observed in mGCs (Fig. 6A). Basal respiration (Fig. 6B) and non-mitochondrial oxygen consumption (Fig. 6F) were significantly increased after FSH stimulation at all concentrations (*P* < 0.05 in the 6 and 600 mIU/ml groups, *P* < 0.01 in the 60 mIU/ml group). ATP production was significantly elevated in the 60 and 600 mIU/ml groups (*P* < 0.05) (Fig. 6D). Maximal respiration (Fig. 6B) and spare respiratory capacity (Fig. 6G) were also significantly increased in cells treated with 600 mIU/ml FSH (*P* < 0.05). No significant differences were observed among all groups in terms of H^+^ leak (Fig. 6E).

### cGCs and mGCs showed different mitochondrial respiration upon FSH exposure

**Figure 7.**
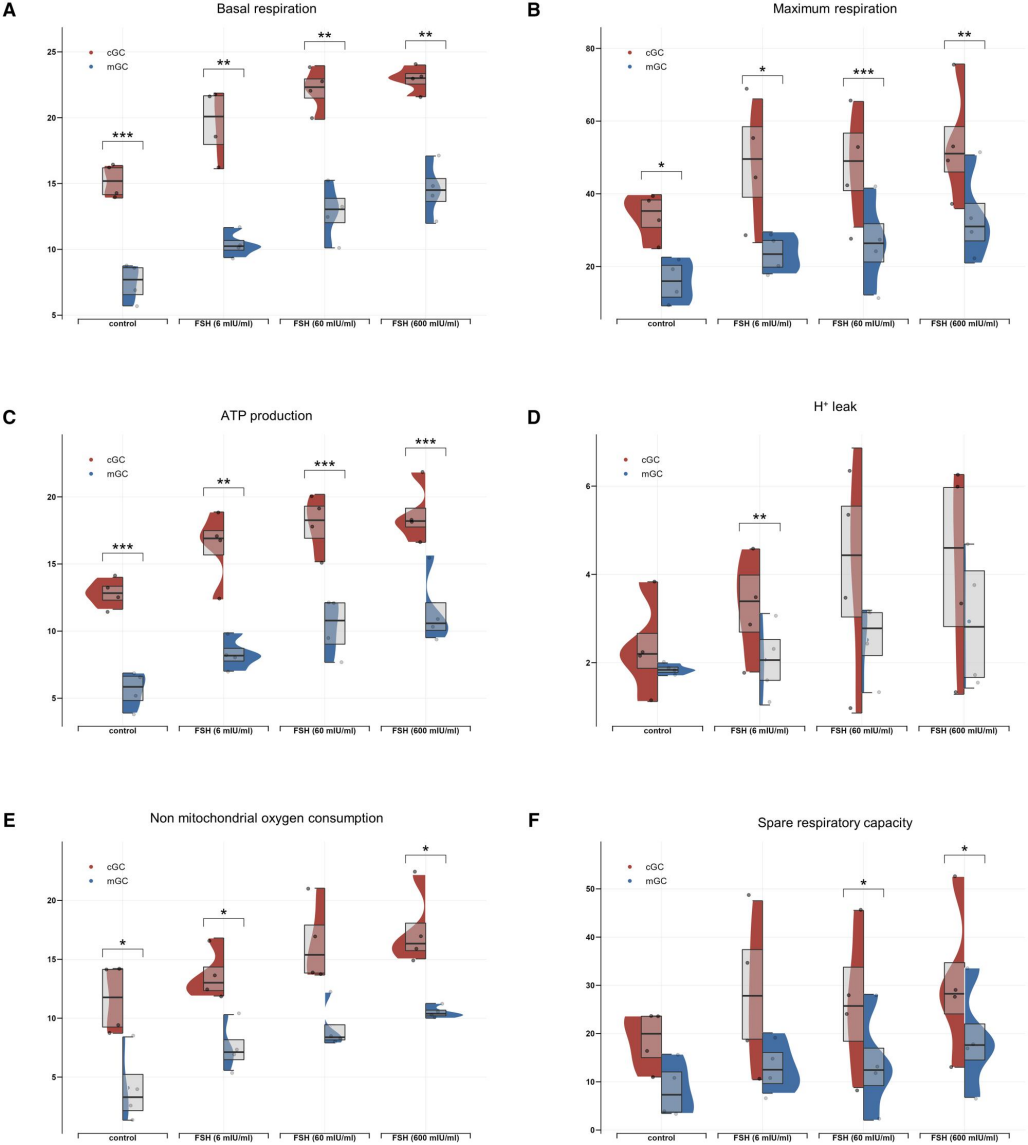
**Differences in mitochondrial respiration between cGCs and mGCs in response to FSH of different concentrations after 2 h**. Differences in mitochondrial respiration, including basal respiration (**A**), maximum respiration (**B**), ATP production (**C**), H^+^ leak (**D**), non-mitochondrial respiration (**E**), and spare respiratory capacity (**F**), were compared between cGCs and mGCs with or without FSH treatments. Significance was tested by using a *t*-test. N = 4 for each group. *P < 0.5, **P < 0.01, ***P < 0.001. cGCs: cumulus granulosa cells; mGCs: mural granulosa cells.

To investigate whether FSH exposure induces different mitochondrial respiration changes between the two GC subtypes, all mitochondrial respiration-related parameters between cGCs and mGCs were compared after 2-h FSH exposure in each group (control, 6 mIU/ml, 60 mIU/ml, 600 mIU/ml). Differences in mitochondrial respiration between cGCs and mGCs with or without FSH stimulation are presented. Compared to cGCs, significantly lower basal respiration (Fig. 7A), maximal respiration (Fig. 7B), and ATP production (Fig. 7C) were observed in all groups in mGCs (*P* < 0.05). mGCs also showed significantly lower basal respiration H^+^ leak (FSH group of 6 mIU/ml, *P* < 0.01) (Fig. 7D), non-mitochondrial oxygen consumption (control group and FSH groups of 6 mIU/ml, 600 mIU/ml, all *P*s < 0.05) (Fig. 7E) and spare respiratory capacity (FSH groups of 60 and 600 mIU/ml, both *P*s < 0.05) (Fig. 7F).

### FSH-induced free radicals do not cause significant oxidative damage in cGCs and mGCs

Low levels of free radicals can function as redox-active signaling messengers, whereas excessive free radicals induce cellular damage (Loor *et al.*, 2010). To investigate whether the increased free radicals upon FSH exposure induce oxidative damage, oxidative damage to lipids, DNA/RNA, and proteins was measured after FSH exposure of different concentrations for 2 h. Menadione, a classical oxidant, was included as positive control since it has been reported to induce toxic oxidative stress associated with mitochondrial DNA damage and cell death (Grishko *et al.*, 2001).

**Figure 8.**
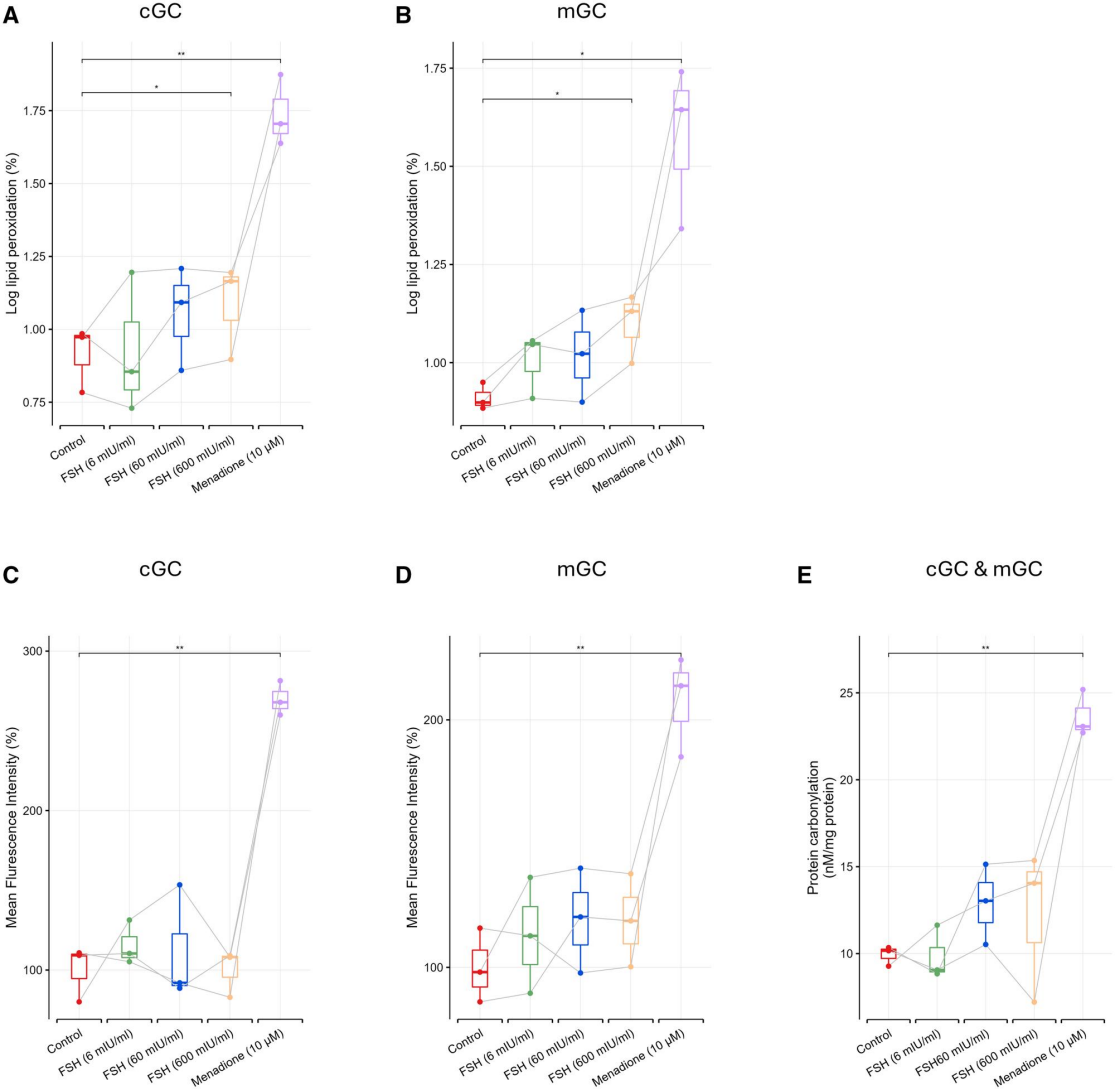
**Oxidative damage to lipids, DNA/RNA, and proteins measured after FSH treatments of different concentrations after 2 h**. Lipid peroxidation was measured by flow cytometry in cGCs (**A**) and mGCs (**B**). Mean fluorescence intensity of DNA/RNA oxidative damage biomarker, 8-OHdG, was measured in cGCs (**C**) and mGCs (**D**) by confocal microscopy. (**E**) Amount of carbonylated proteins were measured by pooling cGCs and mGCs together from three patients of each group. Menadione (10 µM) was used as a positive control. Not all *y*-axes start at 0. Significance was tested by using paired *t*-test. N = 3 for each group. *P < 0.05, **P < 0.01. cGCs: cumulus granulosa cells; mGCs: mural granulosa cells; 8-OHdG: 8-hydroxy-2-deoxyguanosine.

To detect the extent of lipid peroxidation, the classical fluorescent lipid peroxidation probe C11-BODIPY 581/591 was applied. Flow cytometry showed no significantly elevated lipid peroxidation except in the FSH group of 600 mIU/ml (*P* < 0.05) in both cGCs (Fig. 8A, Supplementary Fig. S6) and mGCs (Fig. 8B, Supplementary Fig. S7).

To investigate whether the increased free radicals upon FSH exposure induce DNA/RNA oxidative damage and to explore the potential localization of such damage, the intensity and localization of 8-OHdG were observed and measured. No significant DNA/RNA oxidative damage was found in the FSH groups at any concentration compared to the control, either in cGCs (*P* > 0.05) (Fig. 8C, Supplementary Fig. S8) or mGCs (*P* > 0.05) (Fig. 8D, Supplementary Fig. S9). In all groups, 8-OHdG is absent in nuclei and overlaps with the area where mitochondrial import receptor (TOM20) localizes (Supplementary Figs S8 and S9), indicating that the observed DNA/RNA oxidation was primarily extranuclear.

To investigate whether the increased free radicals upon FSH exposure induce oxidative damage to proteins, protein carbonylation was evaluated. No significant elevation of GC protein carbonylation was found in the FSH groups at any concentration compared to controls (Fig. 8E).

## Discussion

In this study, we used a quantum sensing approach to assess FSH-induced free radical generation in human cGCs and mGCs. This method reveals their different responses on a subcellular level and across cell types on FSH exposure. Furthermore, we found that FSH exposure promoted mitochondrial respiration, a metabolic process during which ROS is mainly produced. Finally, the FSH-induced free radical production does not cause oxidative damage in either cGCs or mGCs, except at supraphysiological concentrations (600 mIU/ml), where it induces lipid peroxidation.

Several existing studies have provided clues on the association between FSH exposure and ROS production with data obtained from mouse models. Chao *et al.* found that oxidative damage to ovarian tissues, without specifying which cells were involved, was positively related to the number of ovarian stimulation cycles using pregnant mare serum gonadotropin (PMSG), an FSH mimic to superovulate female mice (Chao *et al.*, 2005). Another study indicated a higher ROS level in mouse oocytes after PMSG superovulation (Kalthur *et al.*, 2016). Similar to what we have found, Hoque et al. found that ROS levels in mouse GCs were increased following FSH treatment (100 ng/ml, ≈0.6 mIU/ml) for 24 h (Hoque *et al.*, 2021). In contrast, another study pointed out that FSH (7.5 IU/ml) treatment for 2 h can protect H_2_O_2_-primed mouse GCs from oxidative damage *in vitro* (Shen *et al.*, 2016). These seemingly opposing findings can be attributed to the fact that a different downstream signaling cascade is triggered by FSH when GCs are exposed to high levels of ROS. In addition, the duration of FSH exposure varying from 2 h to several cycles of stimulation could lead to differences in results.

Mitochondria are responsible for the majority of ATP generation for energy consumption and thus represent a major source of ROS, which is often a consequence of electron leakage from the electron transport chain (ETC) during mitochondrial oxidative phosphorylation (OXPHOS) (Zorov *et al.*, 2014). Existing literature has underscored the critical role of oocyte and GC mitochondria in oocyte quality, as dysfunction in mitochondrial OXPHOS has been linked to spindle malformation, maternal aging, and decreased oocyte competence (Liu *et al.*, 2017; Sreerangaraja Urs *et al.*, 2020; Adhikari *et al.*, 2022). The temporospatial property of relaxometry allows us to probe free radical formation across cell compartments in real-time upon FSH exposure. As an interesting result, higher free radical generation was observed in mitochondria compared to cytoplasm after 2 h of FSH exposure. This cell compartment discrepancy can be attributed to two factors. Firstly, considering that we also found enhanced mitochondrial respiration (mt-OXPHOS) after FSH exposure, the mitochondria could be the major sites of FSH-induced free radical generation. This idea is further supported by the studies of Hoque et al. They found that the mitochondria play a central role in FSH-induced GC proliferation and differentiation through essential ATP supply throughout mouse follicle development (Hoque *et al.*, 2019). Moreover, the authors revealed that FSH causes oxidative stress and mitochondrial DNA damage, speculating that the excess ROS is derived from high mitochondrial activity and ATP production during the FSH stimulation (Hoque *et al.*, 2021). Another explanation for the differences in cytoplasmic and mitochondrial free radical level change is the compartmentation of ROS types. Superoxide anions ($O_{2}^{•-}$) are the main type of ROS generated in mitochondria, and they can be released from the ETC into both the matrix and the intermembrane space (Nicco and Batteux, 2017). However, due to its charge, superoxide is not expected to appreciably cross membranes into the cytoplasm until being converted to H_2_O_2_ by superoxide dismutase (SOD). Only small amounts of $O_{2}^{•-}$ can cross the outer mitochondrial membrane in the protonated form or pass via anion transporters (Brown and Borutaite, 2012). Relaxometry only detects free radicals, mainly including $O_{2}^{•-}$ and OH^•^ that contain the unpaired electron rather than H_2_O_2_. It is therefore conceivable that aVDAC2-FNDs, which are expected to localize in the outer mitochondrial membrane, detect relatively high levels of $O_{2}^{•-}$ accumulated in the intermembrane space. On the contrary, the cytoplasmic H_2_O_2_ converted from mitochondrial $O_{2}^{•-}$ and leaving the mitochondria will not be detected by bare-FNDs.

Differences between cGCs and mGCs in response to FSH exposure were also found in our study. Although cGCs and mGCs respond to physiological levels of FSH similarly in the aspect of free radical generation, they show different responses to supraphysiological levels of FSH. The percentage of free radical generation was significantly higher in cGCs compared to mGCs. Furthermore, cGCs displayed significantly higher mitochondrial respiration either with or without FSH stimulation in comparison to mGCs. These findings suggest that OXPHOS is more evident in cGCs, and concurrently, more ROS are generated as the by-product. Although no studies on differences in OXPHOS between cGCs and mGCs have been published thus far, a previous study has demonstrated that cultured rat cGCs replicated 10 times more than mGCs, either with or without FSH stimulation (Khamsi and Roberge, 2001). Since cell proliferation requires a constant ATP supply of energy, it is plausible that cGCs are more sensitive to FSH stimulation with higher OXPHOS. Notably, aerobic glycolysis is an alternative pathway to supply ATP for proliferating cells while minimizing intracellular ROS production, thereby protecting against oxidative stress (Brand, 1997). This may explain the finding from another study revealing that transcripts encoding enzymes participating in glycolysis are expressed more by mouse cGCs than mGCs in mouse ovarian follicles (Wigglesworth *et al.*, 2015). Nevertheless, future studies on human cGC and mGC RNA-seq in response to FSH are warranted to examine the underlying pathways.

Excess intracellular ROS production has the potential to adversely affect cells and compromise their function. For instance, the formation of HO* radicals originating from O_2_ can directly damage DNA/RNA, lipids, and proteins (Schofield and Schafer, 2021). The accumulation of ROS-induced damage, associated with mitochondrial dysfunction, was suggested to be one of the mechanisms responsible for ovarian aging (Smits *et al.*, 2023). However, elevated levels of ROS do not inevitably result in oxidative damage, as long as the magnitude of ROS increase does not exceed the capacity of the cellular antioxidant system (Forkink *et al.*, 2015). We found that FSH of all concentrations did not reduce cell viability. This finding was consistent with a previous study where a different assay (Cell Counting Kit-8) was applied (Shen *et al.*, 2016). In addition, FSH did not induce negligible oxidative damage, as only lipid damage at FSH concentrations of 600 mIU/ml was observed. This suggests that antioxidant activities in both GCs are sufficient to prevent ROS-induced oxidative stress and that the ROS generated in FSH-stimulated cells primarily function as redox signaling molecules rather than stressors. Among antioxidants, SOD2 (manganese superoxide dismutase, Mn-SOD) is the principal scavenger of mitochondrial superoxide (Palma *et al.*, 2020). Researchers have found that SOD2 deficiency can lead to oxidative stress, which affects steroidogenesis in mouse GCs (Zaidi *et al.*, 2021). Another important ROS scavenger in the mitochondria is glutathione peroxidase (GPx), which breaks down hydrogen peroxide (H_2_O_2_) (Ighodaro and Akinloye, 2018). It is therefore plausible that the ROS induced by FSH is largely eliminated by SOD2 and GPx so that substantial oxidative damage was detected. It is noteworthy, however, that very high FSH may overwhelm these antioxidant defenses and still has the potential to induce oxidative cellular damage. Although only supraphysiological FSH (600 mIU/ml) induced oxidative damage, its impact should still prompt alertness among clinicians, as increased lipid peroxidation is negatively associated with ovarian response, oocyte maturation, and ART outcomes (Das *et al.*, 2006; Jana *et al.*, 2010; Karakaya *et al.*, 2011; Singh *et al.*, 2013; Kumar *et al.*, 2018; Thaker *et al.*, 2020; Gongadashetti *et al.*, 2021; Tural *et al.*, 2021).

An important advantage of the current study is the application of relaxometry, which allows temporospatial measurements of the free radical change. To date, different methods, either indirect or direct, have been utilized in several studies measuring ROS levels in cumulus and/or mural GCs (Seino *et al.*, 2002; Tural *et al.*, 2021). A combination of traditional direct and indirect methods was also performed in this study: a DCFH-DA assay to measure intracellular ROS, as well as lipid peroxidation assay, protein carbonylation assay, and 8-OHdG detection to quantify oxidative damage to lipid, protein, and DNA/RNA, respectively. Interestingly, while the results from these ROS detection techniques seem not to be completely consistent, they further support the concept that increased ROS does not necessarily act as a stressor; rather, it serves as a redox signaling molecule. The inconsistency also highlights that ROS measurements should be cautiously selected based on the type of ROS of interest (free radicals or non-free radicals, etc.), compartmentation (the whole cell or certain organelles, etc.), and research aim (real-time level or oxidative cellular damage) (Murphy *et al.*, 2022). Although fluorescent dye-based methods have a high sample throughput within a relatively short time, they suffer from the risks of photobleaching over time and reveal the past rather than the current state. Thus, they are not capable of detecting temporospatial changes. The utility of human material represents another advantage of this study, as the existing evidence of FSH on GC ROS stimulation has all originated from mouse studies.

Nonetheless, the present study also has certain limitations. Firstly, the GCs came from females of different biological backgrounds and were stimulated before oocyte and GC retrieval, thereby increasing the risk of variation. For example, the differences in FSH receptor genotype and anti-Müllerian hormone (AMH) level determine variations in FSH sensitivity (Perez Mayorga *et al.*, 2000; Visser and Themmen, 2014). Therefore, the speed and amount of free radical generation after FSH exposure can vary among patients. However, results are very consistent, lending credibility to the study in spite of biological variability. It is also noteworthy that this study only included women possessing a minimum of three follicles on the day of follicle triggering. This means that extremely poor ovarian responders were not included, and the conclusions cannot fully represent the whole population. Secondly, the response of GCs is based on short-term FSH treatment (2 h), so the long-term effects of FSH on ROS generation could be different. Moreover, the *in vivo* effects prior to the granulosa and oocyte retrieval remain a black box since FSH is administered every day at doses between 150 and 225 IU/ml during ovarian stimulation in clinical practice, and the amount of FSH reaching and staying in the follicle is a dynamic mode. Thirdly, the GCs used in this study were luteinized, so their response to FSH could be different from that of unluteinized GCs *in vivo*. However, it is the most feasible option to investigate human GCs. Finally, we still do not know whether the cGCs, which form a protective shield around the oocytes, may transport the FSH-induced ROS to the oocyte itself or that this does not happen. Further research to test the ‘FSH Ootoxicity’ hypothesis should be done on cumulus–oocyte complexes to see whether the ROS production in GCs leads to oxidative stress in the oocyte or not.

In conclusion, this study demonstrates that FSH of both physiological and supraphysiological concentrations induced free radical generation on subcellular levels, most notably in the mitochondria, while the elevated free radical load caused negligible oxidative damage in both cGCs and mGCs. Our results suggest that the ‘FSH Ootoxicity’ hypothesis would not seem to be mediated by ROS in human GCs. Further research on FSH ootoxicity is needed to confirm the hypothesis.

## Supplementary Material

hoaf007_Supplementary_Data

## Data Availability

The data used to support the findings of this study are available from the corresponding author upon request.
