# Supplementary material for: Follicle-stimulating hormone stimulates free radical generation without inducing substantial oxidative stress in human granulosa cells
Source: Hum Reprod Open. 2025 Feb 17;2025(2):hoaf007. doi: 10.1093/hropen/hoaf007 (PMC11893154; doi:10.1093/hropen/hoaf007)
Supplement: hoaf007_Supplementary_Data [file hoaf007_supplementary_data.pdf]

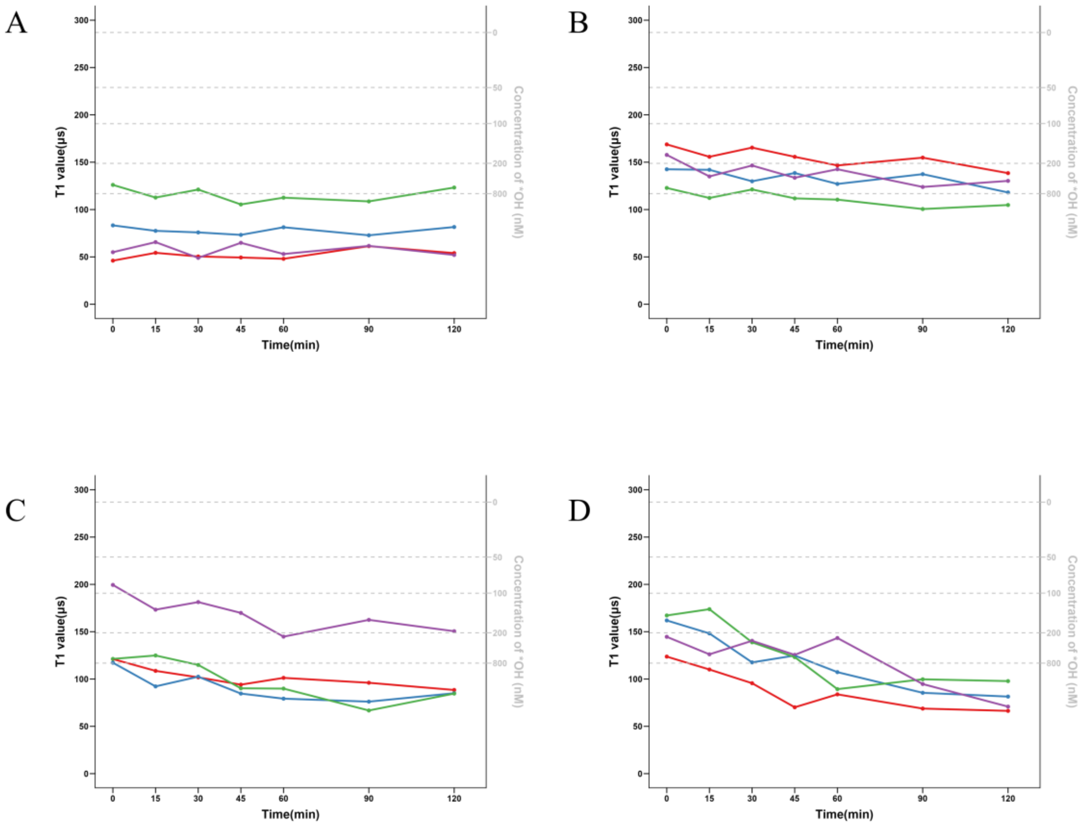

Supplementary Figure S1 T1 raw data obtained from bare FND incubated in cGCs. (A) T1 values were plotted in cGCs without FSH treatment. (B-D) T1 values were plotted in cGCs with different FSH treatments: 6 mIU/ml (B), 60 mIU/ml (C), 600 mIU/ml (D). The right Y axis represents the approximated radical concentration. Each curve represents measurements performed on the same particle at different time.

cGC: cumulus granulosa cells; mGC: mural granulosa cells; FND: fluorescence nanodiamond.

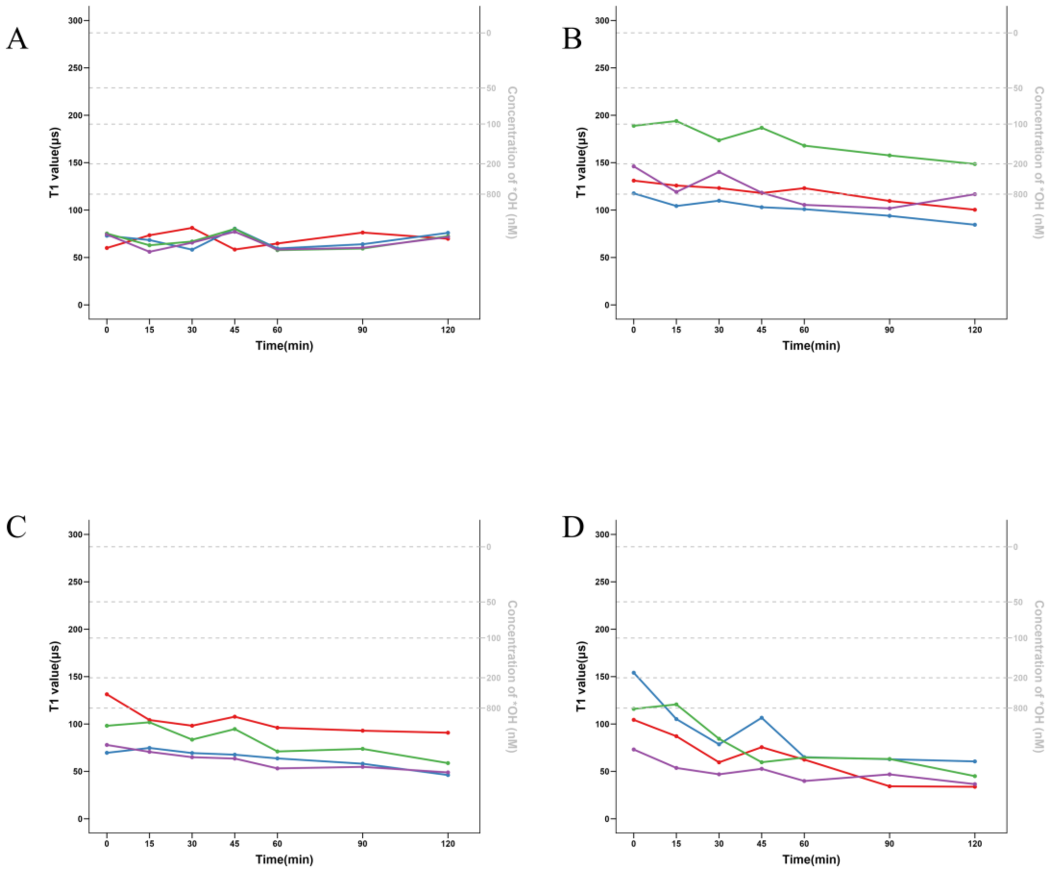

Supplementary Figure S2 T1 raw data obtained from mitochondria- targeting FND incubated in cGCs. (A) T1 values were plotted in cGCs without FSH treatment. (B-D) T1 values were plotted in cGCs with different FSH treatments: 6 mIU/ml (B), 60 mIU/ml (C), 600 mIU/ml (D). The right Y axis represents the approximated radical concentration. Each curve represents measurements performed on the same particle at different time.

cGC: cumulus granulosa cells; mGC: mural granulosa cells; FND: fluorescence nanodiamond.

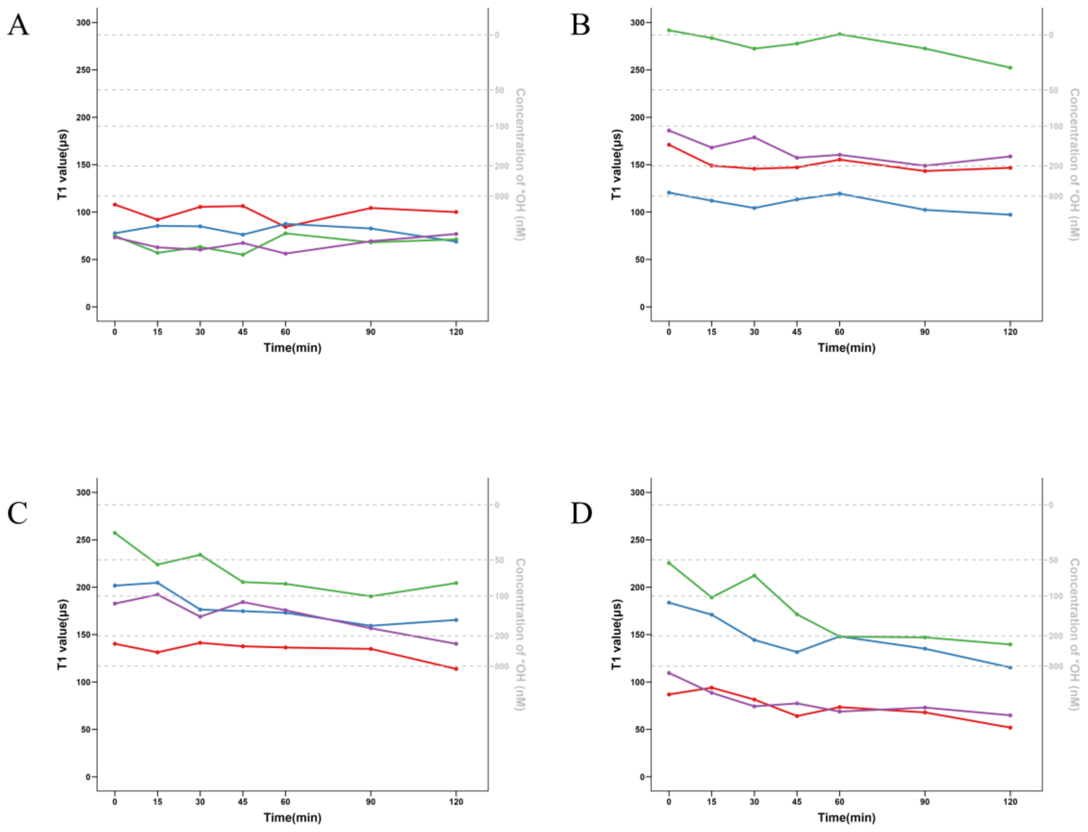

Supplementary Figure S3 T1 raw data obtained from bare FND incubated in mGCs. (A) T1 values were plotted in mGCs without FSH treatment. (B-D) T1 values were plotted in mGCs with different FSH treatments: 6 mIU/ml (B), 60 mIU/ml (C), 600 mIU/ml (D). The right Y axis represents the approximated radical concentration. Each curve represents measurements performed on the same particle at different time.

cGC: cumulus granulosa cells; mGC: mural granulosa cells; FND: fluorescence nanodiamond.

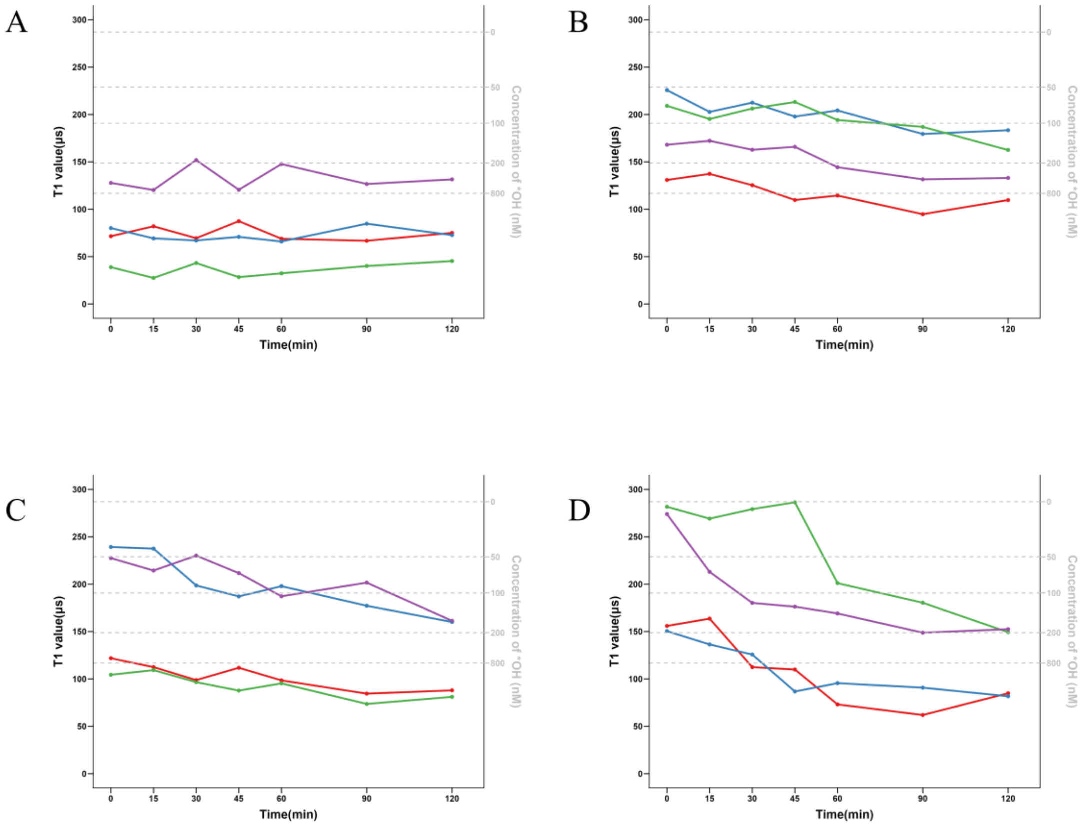

Supplementary Figure S4 T1 aw data obtained from mitochondria- targeting FND incubated in mGCs. (A) T1 values were plotted in mGCs without FSH treatment. (B-D) T1 values were plotted in mGCs with different FSH treatments: 6 mIU/ml (B), 60 mIU/ml (C), 600 mIU/ml (D). The right Y axis represents the approximated radical concentration. Each curve represents measurements performed on the same particle at different time.

cGC: cumulus granulosa cells; mGC: mural granulosa cells; FND: fluorescence nanodiamond.

A

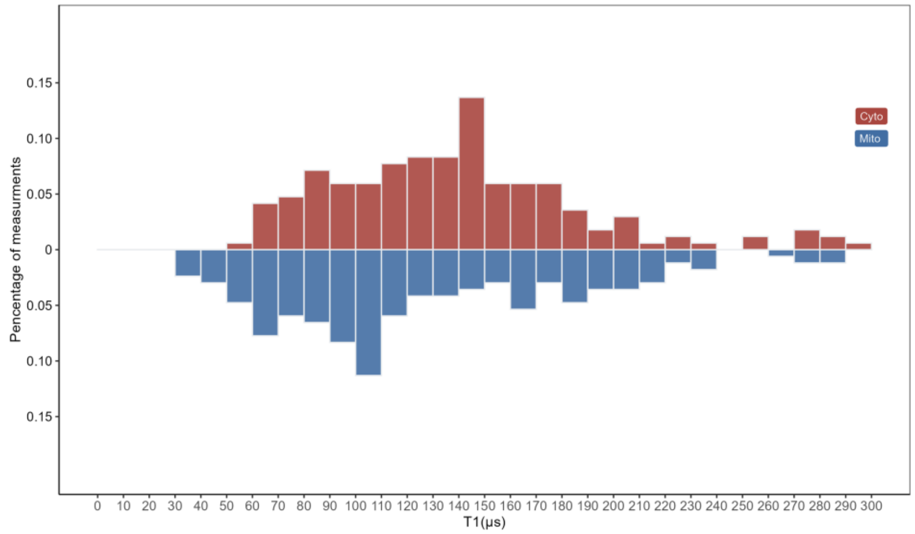

B

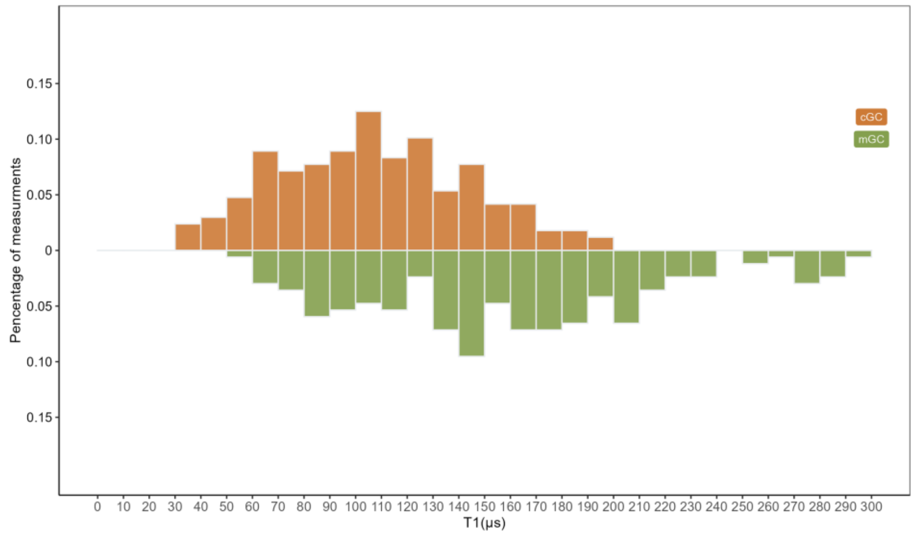

Supplementary Figure S5 T1 histogram of all the measurements. (A) T1 value distributions from bare FNDs and mitochondria- targeting FNDs, respectively in granulosa cells. (B) T1 value distributions from cGCs and mGCs, respectively.

cGC: cumulus granulosa cells; mGC: mural granulosa cells; FND: fluorescence nanodiamond.

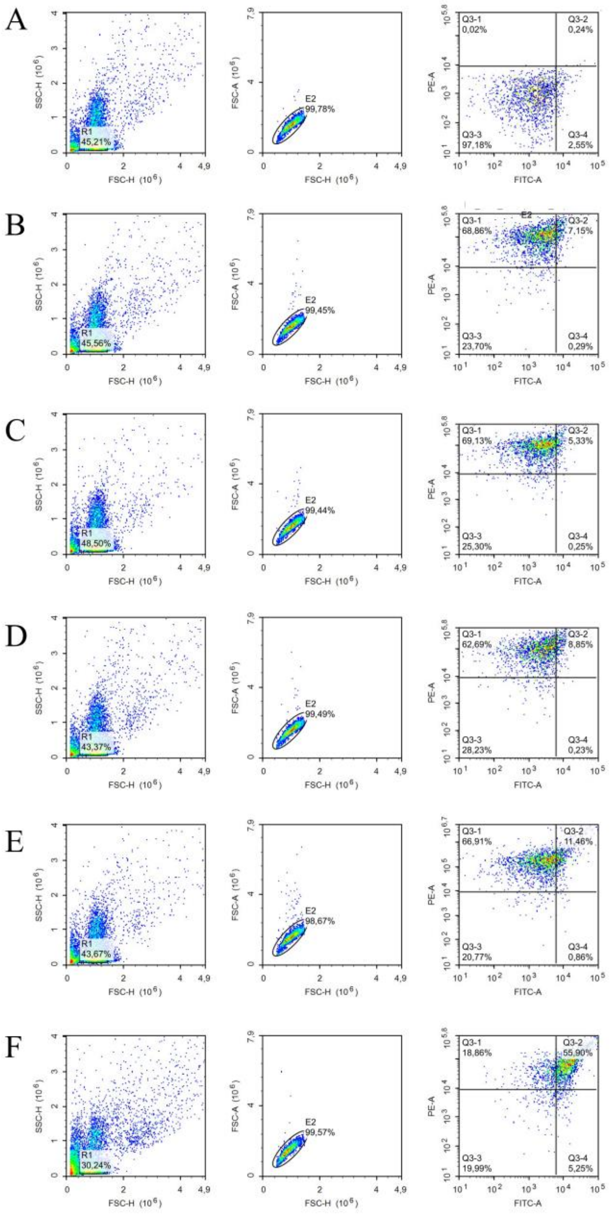

Supplementary Figure S6 Lipid peroxidation of cGCs was assessed by C11-BODIPY staining with flow cytometry 2 hours after FSH treatments. Typical flow cytometry plot and gating in groups of the followings: (A) unstained cGC sample; (B) control; (C) FSH (6 mIU/ml); (D) FSH (60 mIU/ml); (E) FSH (600 mIU/ml); (F) menadione (10 μM).

cGC: cumulus granulosa cells; mGC: mural granulosa cells; FND: fluorescence nanodiamond.

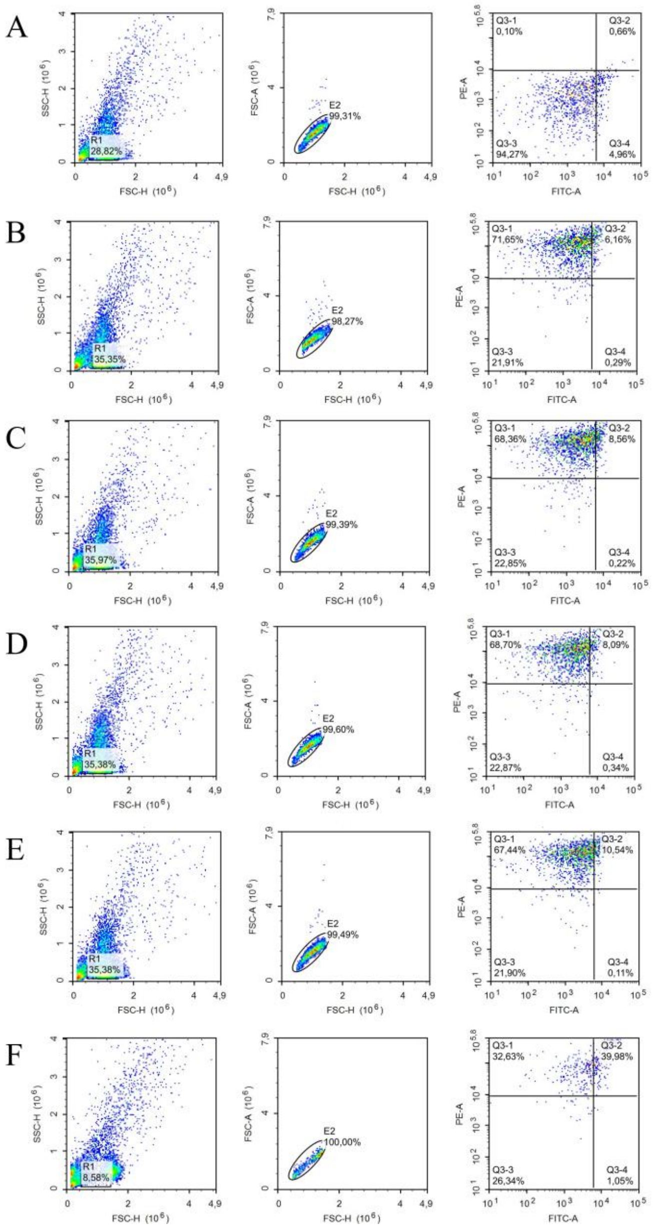

Supplementary Figure S7 Lipid peroxidation of mGCs was assessed by C11-BODIPY staining with flow cytometry 2 hours after FSH treatments. Typical flow cytometry plot and gating in groups of the followings: (A) unstained cGC sample; (B) control; (C) FSH (6 mIU/ml) (D) FSH (60 mIU/ml); (E) FSH (600 mIU/ml); (F) menadione (10 µM).

cGC: cumulus granulosa cells; mGC: mural granulosa cells; FND: fluorescence nanodiamond.

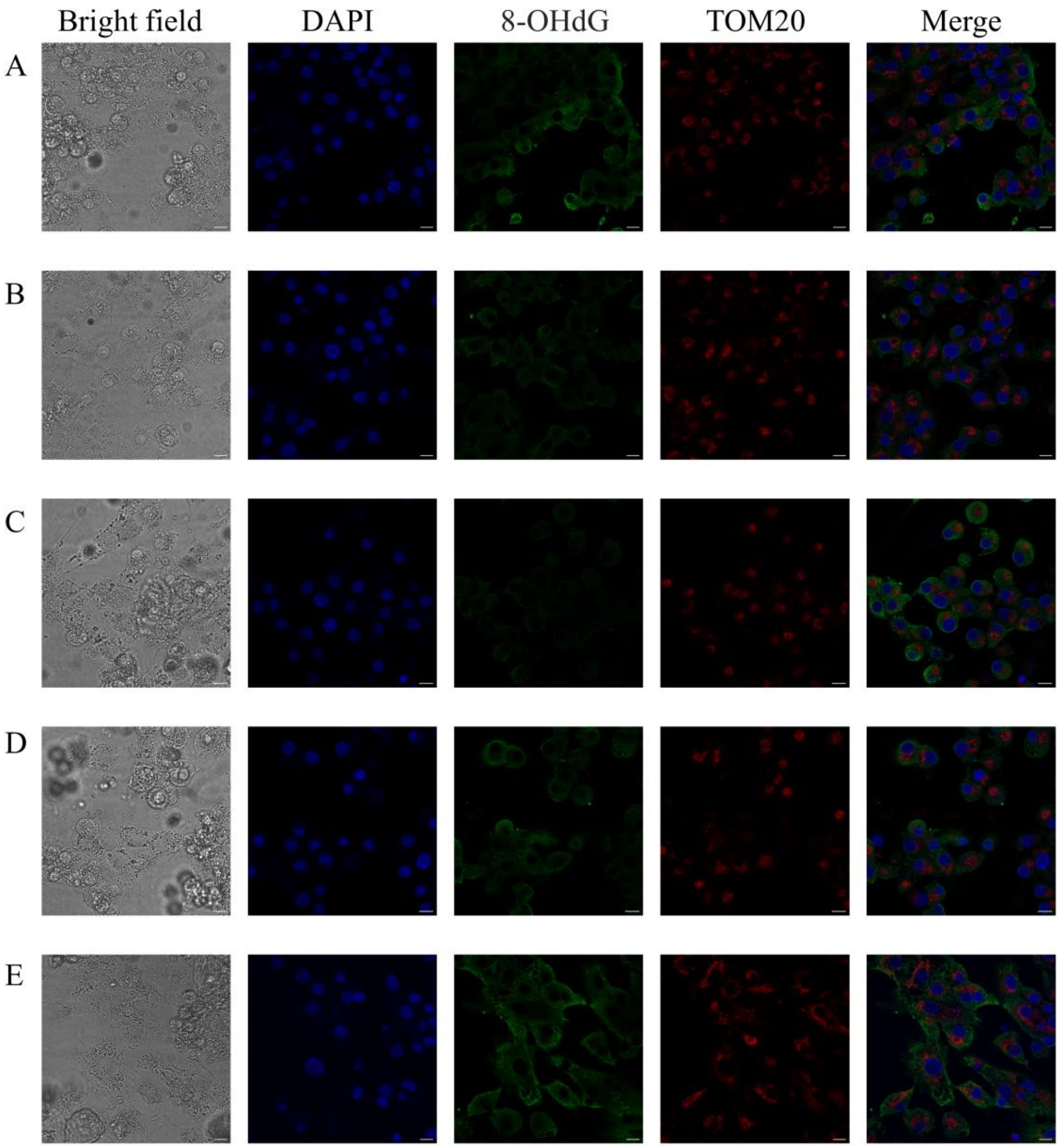

Supplementary Figure S8 Representative images of 8-OHdG expression and distribution in cGCs in groups of the followings: (A) control; (B) FSH (6 mIU/ml) (C) FSH (60 mIU/ml); (D) FSH (600 mIU/ml); (E) menadione (10 μM). Cells were incubated with the anti-8-OHdG antibody followed by a FITC-conjugated secondary antibody. Tom20 antibody, an outer mitochondrial membrane biomarker, was used to show mitochondria. Mean optical intensity in regions of interest were analyzed using FIJI. Color code: green, FITC (for 8-OHdG); red, Red-X (for Tom20); blue, DAPI. The scale bar is 10 μm.

cGC: cumulus granulosa cells; mGC: mural granulosa cells; 8-OHdG: 8-hydroxy-2-deoxyguanosine; FITC: fluorescein isothiocyanate; Tom20: translocase of the outer mitochondrial membrane 20; DAPI: 4′,6-Diamidino-2-Phenylindole.

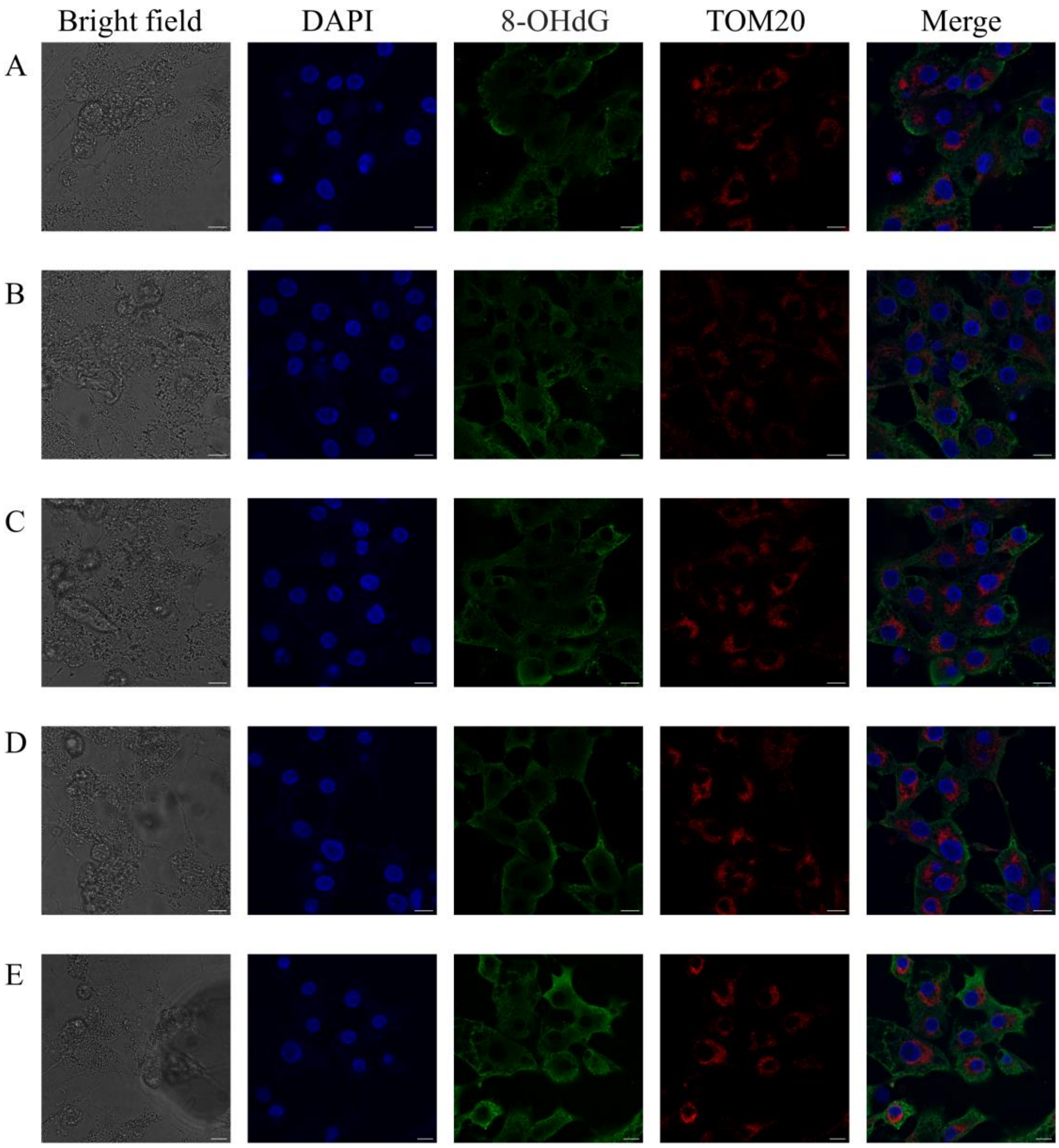

Supplementary Figure S9 Representative images of 8-OHdG expression and distribution in mGCs in groups of the followings: (A) control; (B) FSH (6 mIU/ml) (C) FSH (60 mIU/ml); (D) FSH (600 mIU/ml); (E) menadione (10 μM). Cells were incubated with the anti-8-OHdG antibody followed by a FITC-conjugated secondary antibody. Tom20 antibody, an outer mitochondrial membrane biomarker, was used to show mitochondria. Mean optical intensity in regions of interest were analyzed using FIJI. Color code: green, FITC (for 8-OHdG); red, Red-X (for Tom20); blue, DAPI. The scale bar is 10 μm.

cGC: cumulus granulosa cells; mGC: mural granulosa cells; 8-OHdG: 8-hydroxy-2-deoxyguanosine; FITC: fluorescein isothiocyanate; Tom20: translocase of the outer mitochondrial membrane 20; DAPI: 4′,6-Diamidino-2-Phenylindole.
